# Genetic and Proteomic Evidence for Roles of *Drosophila* SUMO in Cell Cycle Control, Ras Signaling, and Early Pattern Formation

**DOI:** 10.1371/journal.pone.0005905

**Published:** 2009-06-16

**Authors:** Minghua Nie, Yongming Xie, Joseph A. Loo, Albert J. Courey

**Affiliations:** 1 Department of Chemistry and Biochemistry, University of California Los Angeles, Los Angeles, California, United States of America; 2 Department of Biological Chemistry, David Geffen School of Medicine, University of California Los Angeles, Los Angeles, California, United States of America; Gothenburg University, Sweden

## Abstract

SUMO is a protein modifier that is vital for multicellular development. Here we present the first system-wide analysis, combining multiple approaches, to correlate the sumoylated proteome (SUMO-ome) in a multicellular organism with the developmental roles of SUMO. Using mass-spectrometry-based protein identification, we found over 140 largely novel SUMO conjugates in the early *Drosophila* embryo. Enriched functional groups include proteins involved in Ras signaling, cell cycle, and pattern formation. In support of the functional significance of these findings, *sumo* germline clone embryos exhibited phenotypes indicative of defects in these same three processes. Our cell culture and immunolocalization studies further substantiate roles for SUMO in Ras signaling and cell cycle regulation. For example, we found that SUMO is required for efficient Ras-mediated MAP kinase activation upstream or at the level of Ras activation. We further found that SUMO is dynamically localized during mitosis to the condensed chromosomes, and later also to the midbody. Polo kinase, a SUMO substrate found in our screen, partially colocalizes with SUMO at both sites. These studies show that SUMO coordinates multiple regulatory processes during oogenesis and early embryogenesis. In addition, our database of sumoylated proteins provides a valuable resource for those studying the roles of SUMO in development.

## Introduction

Post-translational protein modification adds layers of complexity to macromolecular function. One way of modifying proteins is by joining the ubiquitin family proteins to lysine residues, generating branched proteins [1]. One such ubiquitin-like protein, SUMO (small ubiquitin-related modifier), displays remarkable versatility in modulating target protein function. Many proteins are targeted for covalent modification by SUMO, which consequently modulates many cellular processes [2]–[4].

Genetic analysis has revealed essential roles for SUMO in the survival and development of organisms ranging in complexity from yeast to mammals [2]–[4]. In *S. cerevisiae*, mutations in genes encoding SUMO pathway enzymes are lethal [5]–[7], while mutations in the corresponding genes in *S. pombe* severely impair growth [8]–[10]. Deletion of genes encoding enzymes required for SUMO conjugation in *C. elegans* leads to embryonic lethality [11], while reduction of the SUMO conjugating enzyme levels in *Drosophila*, zebrafish, and mouse results in developmental defects [12]–[14].

The *Drosophila melanogaster* genome encodes a single form of SUMO (herein referred to as *Drosophila* SUMO, but also known as *Drosophila* Smt3), which shares 52% and 73% sequence identity with human SUMO-1 and SUMO-2, respectively [15]. *Drosophila* and human SUMO family proteins are at least partially interchangeable, demonstrating a high level of SUMO pathway conservation between evolutionarily distant organisms [16]. To date, only a few *Drosophila* proteins, such as the transcription factors Dorsal [17], [18], Tramtrack [16], Vestigial [19], SoxNeuro [20], and Medea [21]; the gypsy insulator interacting proteins Mod(mdg4) and CP190 [22]; as well as the bi-functional tRNA charging enzyme glutamylprolyl-tRNA synthetase (EPRS, [23]) are known to be sumoylated. SUMO appears to have diverse roles in the *Drosophila* life cycle, including the regulation of transcription and the modulation of the immune response [18], [20].

While SUMO is present throughout development, early *Drosophila* embryos contain particularly high concentrations of maternally contributed SUMO and the enzymes required for SUMO conjugation [16], [24], [25], suggesting that sumoylation may play particularly critical roles at this stage of fly development. Previous global analyses of SUMO substrates in *S. cerevisiae* and mammalian cultured cells have produced extensive lists of novel sumoylation targets [26]–[35]. To date, however, there are no published studies that document the spectrum of sumoylated proteins in a specific developmental setting in a multicellular organism.

To broaden our understanding of the function of sumoylation in early *Drosophila* development, we performed a mass spectrometry-based global identification of sumoylation targets in early embryos, and found over 140 direct sumoylation targets. Among the identified SUMO target proteins are players in many processes essential to embryonic development, including proteins involved in Ras signaling, cell cycle control, and embryonic patterning. To determine the functional significance of the identified sumoylated proteins, we carried out genetic, cell culture and immunolocalization studies, obtaining evidence for roles of SUMO in these same three processes. Thus, the proteomic, genetic, and cellular studies presented here all converge to suggest that SUMO coordinates key aspects of early metazoan development.

## Results

### Isolation, identification, and categorization of early embryonic SUMO conjugates

**Figure 1:**
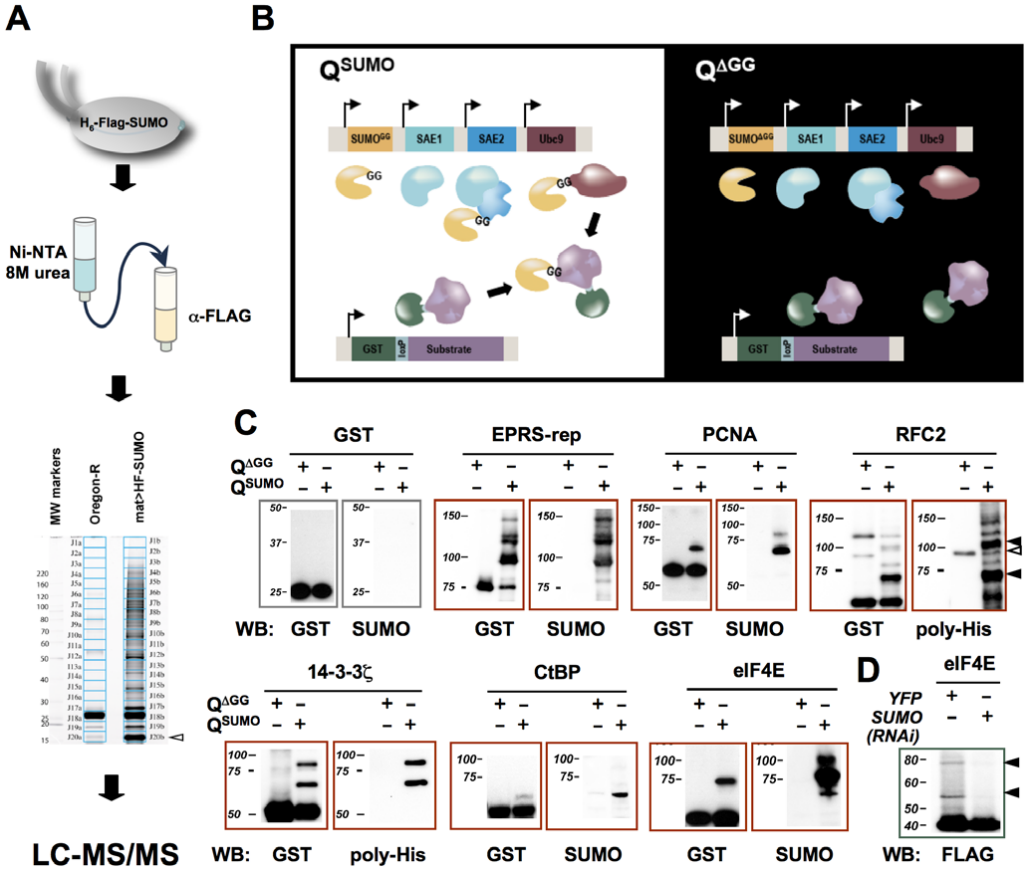
A fly SUMO-ome: characterization and validation. A) Scheme for identifying *Drosophila* embryonic SUMO conjugates. SUMO conjugates were isolated by tandem affinity purification from transgenic fly embryos expressing (His)_6_-FLAG-SUMO. The initial purification step (Ni-NTA chromatography) was performed under denaturing conditions. To maximize the chance of detecting low abundance proteins in the complex protein mixture, the affinity-purified proteins were separated by SDS-PAGE, and the lane was cut into 20 evenly divided gel slices. Tryptic peptides extracted from each gel slice were analyzed by LC-MS/MS. B) A bacterial sumoylation assay. The Q^SUMO^ vector, which encodes the mature form of SUMO (SUMO^GG^) along with SAE1, SAE2, and Ubc9 expressed from separate *T7/lac* promoters, was used in combination with a vector expressing a GST-tagged candidate substrate. As a negative control, Q^ΔGG^, which expresses a conjugation defective form of SUMO (SUMO^ΔGG^), was used in place of Q^SUMO^. C) Bacterial sumoylation assays were used to validate proteins identified in the proteomic screen as sumoylation substrates. GST-tagged candidate SUMO conjugates were expressed in BL21 cells co-transformed with Q^SUMO^ or Q^ΔGG^ vectors, purified using glutathione beads, and immunoblotted using antibodies against GST, SUMO, or poly-His (to detect 6xHis-tagged SUMO). GST by itself was not sumoylated in this assay. Black arrows point to the bands representing sumoylated proteins, and open arrow points to a non-specific reacting band. D) The eIF4E protein was purified from *Drosophila* S2 cells stably expressing FLAG-(His)_6_- tagged eIF4E using Ni-NTA beads under denaturing conditions. The resulting proteins were probed with anti-FLAG antibody in a Western blot. The cells were treated with SUMO or control YFP dsRNA for 3 days prior to cell lysis. In the control sample, the bands representing the sumoylated species (black arrows) have intensities that are 8.1% (top) and 12.9% (bottom) of the intensity of the band representing unmodified eIF4E (∼40 kDa), whereas in the SUMO knockdown sample, they are reduced to 1.8% (top) and 3.5% (bottom). Quantitation was performed using Quantity One 4.3.0 (BioRad).

To determine the early embryonic SUMO-ome (catalog of sumoylated proteins), we adopted a scheme that involved a two-step affinity purification strategy using SUMO tagged at its N-terminus with both (His)_6_ and FLAG tags, followed by liquid chromatography-tandem mass spectrometry (LC-MS/MS)-based protein identification of trypsin-digested proteins (Figure 1A). We initially attempted to express tagged SUMO using modified *sumo* genomic clones, but were unsuccessful presumably due to the need for unknown distant cis-regulatory modules to direct *sumo* expression. We therefore turned to the Gal4-UAS system [36], and drove ubiquitous maternal expression of tagged SUMO at levels slightly lower than that of endogenous SUMO (Figure S1). Tagged SUMO rescues the lethality resulting from *sumo* mutations (data not shown) demonstrating the functionality of the tagged protein.

In other organisms and cultured cells, heat shock is known to enhance global sumoylation [34], [37]. We observed a similar phenomenon in early embryos (Figure S1). Since the SUMO conjugates were in low abundance even when very large amount of starting materials (five grams of fly embryos) were used, we promoted SUMO conjugation with heat treatment at 37°C. As a control, wild-type Oregon-R (Ore-R) embryos lacking tagged SUMO were collected and processed under identical conditions. While the use of heat shock could raise a concern about the possibility of skewing the protein pool, our pilot study in which proteins were isolated from heat-shocked and non-heat-shocked samples revealed similar SDS-PAGE profiles (Figure S2). Furthermore, analysis by LC-MS/MS revealed a largely overlapping set of proteins from heat-shocked and non-heat-shocked embryos (supporting document S1 and Table S3). The observed differences were largely quantitative rather than qualitative (Table S3)–more peptides were identified from the majority of the proteins in the heat-shocked than in the non-heat-shocked samples, leading to higher confidence protein identification. Moreover, the consistency between our phenotypic analysis (see below) and our proteomic data further increases our confidence in the biological relevance of our SUMO-ome.

In the first step of the two-step affinity purification, the (His)_6_ tag was bound to nickel-coupled agarose under strongly denaturing conditions (containing 1% CHAPS, 8 M urea) in order to solubilize proteins from all cellular compartments, suppress SUMO isopeptidases, and ensure that the purified proteins are directly conjugated to SUMO. To achieve a higher degree of purification, second affinity chromatography step employing anti-FLAG antibodies was carried out. Sypro Ruby staining of a gel with proteins purified from embryos containing dual-tagged SUMO and from negative control embryos, demonstrated very few proteins in the control sample (Figure 1A). The SDS-PAGE gel lanes containing the control and experimental samples were each cut into 20 equal-size slices (Figure 1A), followed by in-gel tryptic digestion and subsequent analysis by LC-MS/MS.

LC-MS/MS data were analyzed using Mascot (Matrix Science) to search the database of known *Drosophila* protein sequences. For proteins represented by four or fewer peptides, we manually inspected the mass spectra to confirm the protein identifications. SUMO tryptic peptides were detected in every gel slice from the sample prepared from embryos expressing tagged-SUMO, and in none of the gel slices from the sample prepared from control Ore-R embryos. A total of 144 proteins (corresponding to 142 genes) were uniquely found in the sample from embryos expressing tagged-SUMO (Table S1). In addition to a large number of novel sumoylation substrates identified, this list includes nearly all previously validated *Drosophila* SUMO conjugates that are present at this developmental stage (e.g., Dorsal, Mod(mdg4), CaMKII, EPRS), as well as proteins that are orthologous to well-characterized SUMO substrates found in other organisms (e.g., PCNA, CtBP, Topoisomerase I and II), thus adding to our confidence in the authenticity of our SUMO-omic database.

In a separate study, tagged SUMO conjugates were isolated under native conditions in a single-step anti-FLAG immunopurification, and over 300 gene products were identified (Table S2). This set includes a large fraction of the proteins (∼60%) identified in the two-step purification procedure (marked by “*” in Table S1). The native purification also appears to have isolated a number of proteins that interact with the SUMO conjugates identified in the two-step purification carried out under denaturing conditions, as well as proteins that were later demonstrated to be authentic SUMO conjugation targets by independent validation methods (see below).

We used a SUMO conjugation site prediction algorithm, SUMOsp [38], to analyze the proteins identified in the one- and two-step purifications (Table 1). While the proteins in the entire *Drosophila* proteome contain an average of 0.76 consensus SUMO conjugation sites per protein, the proteins from the two-step purification average 1.46 consensus sites per protein, and the proteins from the one-step purification average 1.06 consensus sites per protein. These differences are highly statistically significant (Table 1). The lower number of sites in the proteins from the one-step purification relative to the two-step purification further supports the idea that the one-step purification yielded a mixture of direct SUMO conjugation targets and their interacting partners.

**Table 1:** Predicted SUMO consensus sites.

Dataset	Number of Proteins	Number of Sites*	Sites per Protein	Site per aa (×1000)	*p*-value#
Two-step purification	144	210	1.46	2.17	1.48e-06
One-step purification	247	262	1.06	2.03	2.82e-04
*Drosophila* proteome	32182	24886	0.77	1.58	-

* Proteins from the two-step or one-step purification protocols, or total *Drosophila melanogaster* proteins (as annotated in the UniProtKB database) were analyzed using the SUMOsp program to yield the total number of SUMO conjugation sites that match the canonical ψKxE/D consensus motif for each dataset.

# *p*-values were calculated using the hypergeometric distribution to compare the number of predicted sites per amino acid in the databases of purified proteins to the number of sites per amino acid in the *Drosophila* proteome.

We used the Generic Gene Ontology (GO) Term finder tool (http://go.princeton.edu/cgi-bin/GOTermMapper) to search for overrepresented GO categories in the SUMO substrate list when compared to the entire fly proteome. Using the hypergeometric distribution analysis, we calculated the probability that the proportion of SUMO substrates mapped to a given category could occur by random chance, given the fraction of all fly proteins that map to that category (Table S4, S5). This analysis helped us to distinguish several enriched functional groups of SUMO conjugates (Table 2). We also analyzed the enrichment of biological process GO terms in our SUMO proteome when compared to the early *Drosophila* embryonic proteome (2 hr AEL embryo [39], [40]) (Table S6). Many of the same categories that are enriched in comparison to the entire *Drosophila* proteome are also significantly enriched in comparison to the early embryonic proteome.

**Table 2:** Enriched functional groups of sumoylation substrates.

***Cell Cycle Process***
Asp, Awd*, Cup*, Young arrest, Hsp83*, Polo * †, Pumillio, Topoisomerase 2*, Valois, Pp1-87B*, Cdc2c*, PP2A catalytic subunit (Microtubule star) * †, αTub67C*, 14-3-3 ζ *, PCNA * †, RFC2 * †, Twinstar*, Pitslre, Rpn2*, Klp10A*, Brat, (RFC1)#
***Embryonic Pattern Formation***
Bicoid, Caudal, Dorsal, Hunchback, Hsp83*, Osa, Pumillio, Retained
***Maternal mRNA Regulation***
Pumilio, Cup*, Brat, Smaug, Bicoid, Tsunagi, Mago Nashi*, Hrb27C*, NACα, Hel25E*, Valois, Me31b*, (Vasa, eIF4E †, Squid †)#
***Ras1 signaling pathway***
PP2A 65kD subunit A*, PP2A catalytic subunit (Microtubule Star) * †, 14-3-3 ε*, 14-3-3 ζ * †, Hsp83*, (PP2A regulatory subunit Twins, Ras1 †, ERK-A, Phyllopod)#
***Epigenetic regulation***
Osa, Pho, Su(Var)3-7*, Mod(mdg4), H2Av*, Nlp*, Caf1*, CtBP *, (H2B, Rpd3, Groucho, Mi-2, HP1)#
***Nucleocytoplasmic transport***
Exportin-1*, Importin-α re-exporter*, Importin β*, Importin-α*

* These proteins were identified through both the two-step purification and the single-step purification.

# Proteins in parenthesis were identified through the one-step purification only, and therefore may not be direct sumoylation targets. All other proteins were identified through the two-step purification or through both the two-step purification and the single-step FLAG IP.

† These proteins were validated through the bacterial sumoylation assay as SUMO substrates are underlined. A subset of these assays is shown in Figure 1.

Inspection of the list of sumoylated proteins also suggests that protein complexes, such as the *oskar* mRNP, multi-aminoacyl-tRNA synthetase complex, a PCNA-containing protein complex, and the ribosome, often contain multiple sumoylated proteins (Table 3).

**Table 3:** Protein complexes that include multiple sumoylation substrates.

***osk mRNP***
Mago Nashi*, Tsunagi, Cup*, Me31b*, Hrb27C*, NACα, Smaug, Hel25E*, Valois, (eIF4E, squid, Vasa)#
***PCNA***
PCNA*, RFC2*, Caf1*, (DNApol-δ, DNApol- δ small subunit, RFC1)#
***tRNA Multi-Synthetase Complex (MSC)***
EPRS*, QRS*, RRS*, (p38)#
***Protein Phosphatase 2A (PP2A)***
65kD subunit A*, catalytic subunit Microtubule Star*, (regulatory subunit Twins)#
***26S Proteasome***
regulatory subunits: Dox-A2*, Rpn2*, Rpn7*, (Rpn11)#
***Ribosome Complexes***
RpS4*, RpL8*, RpS10b, RpL27A*, RpS7*, RpS3A*, RpS16*, RpS19*, RpL13*, RpL9*, RpS3*, RpL10Ab*, (RpS26, RpS6, RpS10a, RpL28, RpS13, RpL13A, RpS14a, RpS20, RpL15, RpL31, RpS15Aa, RpL7A, RpS9, RpL23, RpS17, RpL7, RpL4, RpL14, RpL18A, RpS25, RpL22, RpLP0, RpL36, RpL11, RpS8, RpL32, RpS23, RpS18, RpS5a)#

* These proteins were identified through both two-step and one-step purification procedures.

# Proteins in parenthesis were found through the one-step purification only, and therefore may not be direct sumoylation targets.

### Validation of SUMO conjugates found through global MS analysis

Using a bacterial sumoylation assay we validated a number of the proteins in the enriched groups as SUMO conjugation targets (Figure 1B). For this assay, we generated *E. coli* expressing the four *Drosophila* polypeptides essential for SUMO conjugation (SAE1, SAE2, Ubc9, and SUMO) as well as a GST-tagged candidate SUMO substrate. The detection of SUMO conjugates in this system is facilitated by the lack of an absolute requirement for E3-type ligases in sumoylation [41], [42] as well as by the absence of SUMO deconjugating enzymes in bacteria. While bacterial sumoylation systems may not completely recapitulate the specificity of sumoylation in vivo, they have been repeatedly validated as a useful approach for confirming sumoylation targets [43]–[46].

Our bacterial sumoylation system includes several improvements over the existing bacterial systems. We use a single vector (Q^SUMO^) to encode all four polypeptides required for sumoylation, thereby reducing variation in expression levels. In addition, we incorporated a *loxP* site into a GST fusion protein expression vector to enable high-throughput cloning of cDNAs encoding potential sumoylation targets. To provide a control for specificity, we generated a control vector, Q^ΔGG^, which encodes a conjugation defective form of SUMO lacking the C-terminal di-glycine motif required for SUMO conjugation [6], [23].

Using this system we confirmed sumoylation of PCNA, the processivity factor for DNA polymerase δ, and RFC2 (Figure 1C), a subunit of the factor that loads PCNA onto DNA. We also verified sumoylation of 14-3-3 ζ (Figure 1C), which belongs to a family of small proteins that interact with a multitude of functionally diverse signaling proteins by binding to phosphorylated serine or threonine residues [47]. *Drosophila* 14-3-3 ζ has been shown to function both in the Ras/MAPK pathway and in regulation of the nuclear cleavage cycles in the syncytial embryo [48]. Using the same assay system, we also confirmed that EPRS, CtBP, eIF4E (Figure 1C), Squid (Figure S4B), and several other proteins found in our MS identification (proteins underlined in Table 2) are sumoylation substrates. We also showed that eIF4E is sumoylated in S2 cell culture, further confirming it as a genuine SUMO substrate (Figure 1D).

This bacterial sumoylation system does not non-specifically sumoylate any substrate as shown by the negative controls. In addition to GST, we also did not detect sumoylation of GFP or HP1 (Figure S4A). This latter protein was identified in the single-step native purification, but not through the two-step protocol that involved initial denaturation of the extract. This suggests that HP1 interacts with sumoylated proteins, but is not itself a sumoylation substrate. In addition, using this assay, we have been able to map specific sumoylation sites in a number of proteins including EPRS, Grauzone, Meics (data not shown), and Ras1 (see below).

### Maternally contributed SUMO is required for embryonic development

Among the enriched functional groups from the proteomic screen (Table 2) are groups of proteins with functions in embryonic pattern formation, including transcription factors, such as Dorsal, Bicoid, and Hunchback, that guide the dorsoventral or anteroposterior patterning of the embryo, as well as proteins involved in the localization and translational regulation of important maternal transcripts. We also found significant enrichment of proteins involved in cell cycle regulation and the Ras signaling pathway. To determine if these enriched functional groups reflect the roles of SUMO in *Drosophila* embryonic development, we carried out genetic and phenotypic analysis of flies carrying hypomorphic *sumo* alleles.

**Figure 2:**
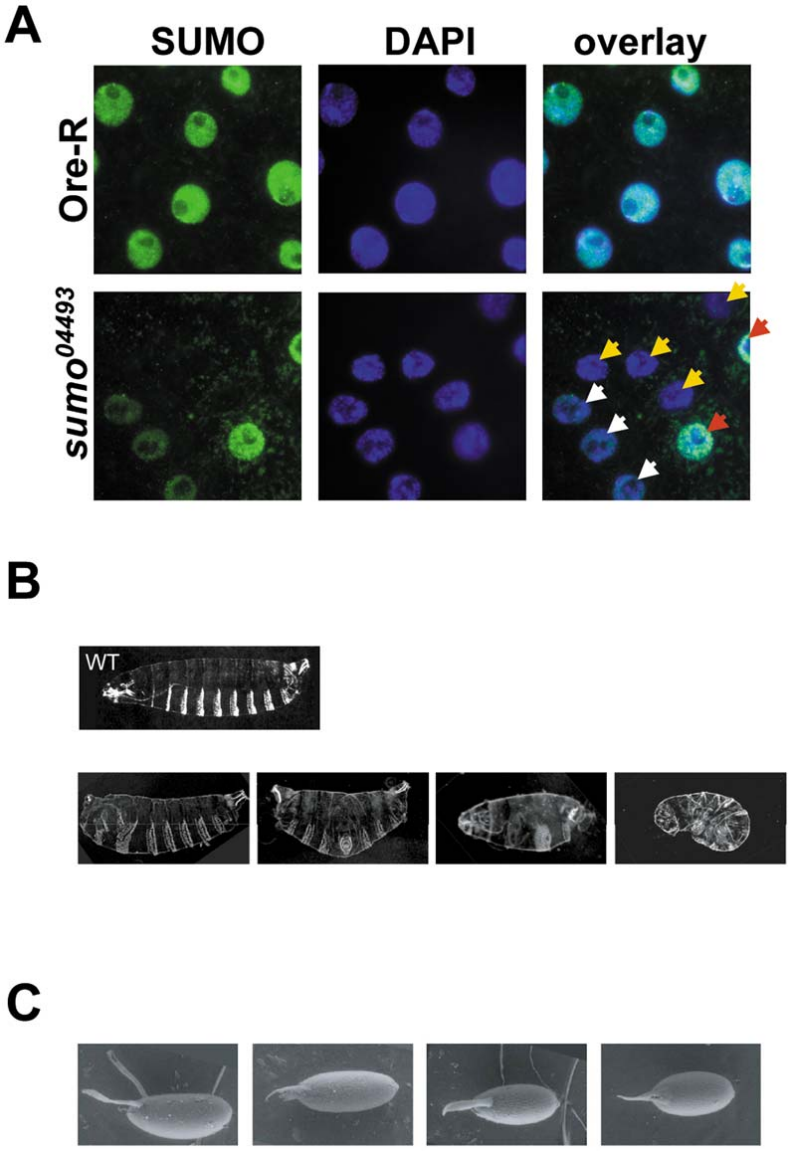
Roles for SUMO in embryonic and eggshell patterning. A) Follicle cell clones in the *sumo^04493^* GLC egg chambers. Wild-type (Ore-R; top) and *sumo^04493^* GLC egg chambers (bottom) were stained with SUMO antibodies (green) and DAPI (blue). The field in the lower panels contains a *sumo^04493/04493^* clone resulting from FLP/FRT recombination (yellow arrows), heterozygous *sumo^04493/+^* cells with a reduced level of SUMO (white arrows), and a *sumo^+/+^* twin spot containing a level of SUMO comparable to that observed in Ore-R follicle cells (red arrows). B) A variety of anteroposterior (3 panels on the left) and dorsoventral (panel on the right) patterning defects were observed in *sumo^04493^* mutant GLC embryos. Among the embryos that formed cuticles, 59% (n = 171) exhibited abnormal cuticular morphology. Top panel shows a wild type cuticle. C) Examples of ventralized eggshells of *sumo^04493^* GLC embryos are shown in the three panels on the right. A wild type Ore-R embryo is shown in the leftmost panel.

Two independent *P*-insertions *sumo* alleles, termed *sumo^04493^* and *sumo^k01211^*, were subjected to phenotypic analysis. The *P*-insertion in both alleles is 20 bp upstream of the transcription start site, creating recessive lethal mutations with a lethal period before or during the early second larval instar. Evaluation of mRNA levels by RT-PCR in homozygous mutant larvae shows that the *sumo^04493^* mutation leads to an approximately 5-fold decrease in the level of *sumo* transcripts (Figure S3). Antibody staining of mutant follicle cell clones (see below) also demonstrates a significant reduction in SUMO levels in mutant tissue (Figure 2A). *sumo^04493^*, *sumo^k01211^*, and a *sumo* EMS allele (generated by Shanti Chandrashekaran, New Delhi, and obtained through Dr. Lawrence Marsh), which contains a serine-glycine sequence in place of the normal C-terminal di-glycine motif required for efficient conjugation of SUMO to its targets [6], [23] fail to complement one another.

To determine the function of maternally contributed SUMO, we created females containing homozygous *sumo* mutant germline clones (GLCs) using the hsFLP/FRT dominant female sterile method [49]. The two *P*-insertion alleles of *sumo* exhibited overlapping spectra of defects, with the average severity of the defects being greater in the *sumo^k01211^* GLC embryos (Table 4). Since the *sumo^k01211^* GLC embryo-producing females laid relatively few mature eggs, most of the subsequent phenotypic analysis was carried out using the *sumo^04493^* GLC embryos.

**Table 4:** Summary of phenotypes observed in *sumo* mutant GLC embryos.

	*sumo^k01211^*	*sumo^04493^*
Defective eggshells	30% (n = 171)	10% (n = 400)
Hatching rate	0% (n = 171)	23±0.9% (n = 800)
Unhatched embryos forming cuticle	0%	10% (n = 200)
Cuticles formed that appear wild-type	–	41% (n = 171)

Less than 30% of the *sumo^04493^* GLC embryos hatched, and all hatched larvae died during the first larval instar. Greater than 90% of the unhatched embryos died prior to cuticle formation. The embryos that deposited cuticle exhibited a wide range of both anteroposterior and dorsoventral patterning defects (Figure 2B). These defects are not rescued by zygotic SUMO expression since embryos produced by GLC females mated with wild-type males had similar high rates of lethality and a comparable spectrum of defects as those embryos produced by GLC females mated with heterozygous males. Both the patterning defects in the *sumo* mutant GLC embryos and the large number of SUMO targets from our proteomic analysis with roles in embryonic pattern formation support the conclusion that SUMO modulates the activities of key pattern formation gene products to help direct embryonic development.

### SUMO plays roles in eggshell patterning and potentiates Ras/MAPK signaling

Approximately ten percent (39 out of 400) of the *sumo^04493^* GLC eggs exhibited partially to fully fused dorsal appendages, indicative of weak to moderate eggshell ventralization (Figure 2C). *Drosophila* eggshell patterning is regulated by the epidermal growth factor receptor (EGFR) signaling pathway. EGFR, a transmembrane receptor tyrosine kinase (RTK), is found in the follicle cells where it receives a spatially localized signal from the developing oocyte. This signal activates the Ras signaling cascade, which patterns the follicle cell epithelium, and is therefore essential for proper patterning of the eggshell [50]–[52]. Both *sumo^04493^* and *sumo^k01211^* have been shown to enhance the weakly ventralized eggshell phenotypes of a hypomorphic *Ras1* mutant [53]. Our observation that the *sumo* mutation leads to eggshell ventralization even in a wild-type *Ras1* background further supports a role for SUMO in EGFR/Ras signaling.

Previous epistasis studies showed that reduced SUMO levels suppressed the eggshell dorsalization resulting from a constitutively active form of EGFR, indicating that SUMO acts downstream of EGFR in the follicle cells [53]. Since the process of generating GLC also leads to production of somatic clones in the follicle cells (Figure 2A), our findings are consistent with a role for SUMO downstream of EGFR in eggshell patterning. Intriguingly, our proteomic analysis also found multiple proteins involved in Ras signaling downstream of EGFR activation as potential SUMO targets (Table 2). Further evidence that SUMO functions downstream of EGFR is provided by experiments described below in which we examine the effect of SUMO knockdown on Ras signaling in S2 cells.

**Figure 3:**
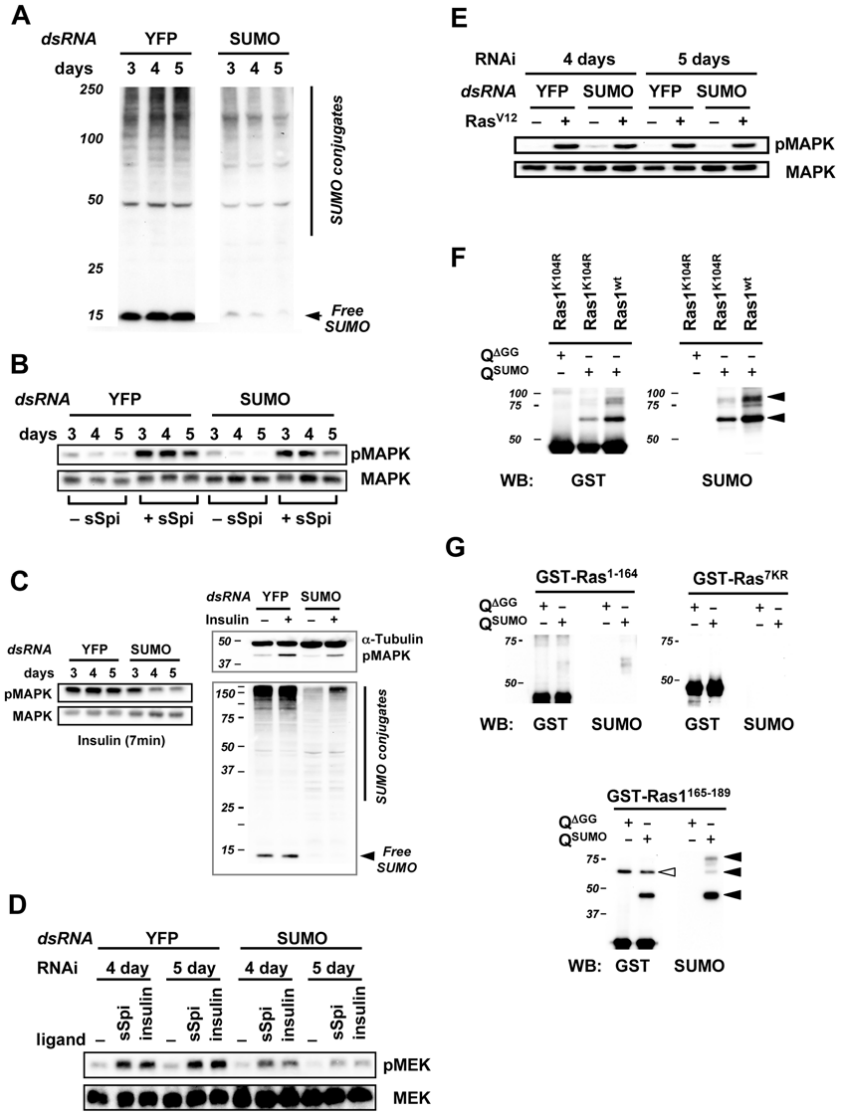
SUMO is required for Ras/MAPK signaling. In panels A to E, lysates from equal number of cells were loaded onto SDS-PAGE. A) Anti-SUMO immunoblot of total protein from cells treated with SUMO or control YFP dsRNA. B, C) EGFR expressing S2R+ cells were treated with YFP or SUMO dsRNA for 3 to 5 days. At the end of the RNAi treatment, 2×10^6^ cells were incubated in serum-free medium for one hour, then exposed to secreted Spitz (sSpi) ligand or insulin for 7 min and immediately lysed for Western analysis. The blots were probed with antibodies against double-phosphorylated MAPK (pMAPK) or total MAPK in B and in left half of C. In the right half of panel C, S2 cells treated with dsRNA for 5 days were stimulated with insulin, and the same samples were probed with antibodies against α-Tubulin, pMAPK, or SUMO. D) An immunoblot of cells treated with YFP or SUMO dsRNA, and exposed to sSpi or insulin, was probed with antibodies against phosphorylated MEK (pMEK) or total MEK. E) An immunoblot of Ras^V12^-expressing S2 cells treated with YFP or SUMO dsRNA was probed with antibodies against pMAPK or total MAPK. Ras^V12^ expression was induced with copper during the last 18 hr of dsRNA treatment. F) Bacterial sumoylation of GST-Ras1 and Ras1^K104R^ using the approach described in the legend to Figure 1. G) Bacterial sumoylation of GST-Ras1^1–164^, Ras1^7KR^, or Ras^165–189^. In the lower panel (GST-Ras^165–189^), the black arrowheads point to the bands representing sumoylated proteins. Based on size, these are likely the mono-, di-, and tri-sumoylated species. The di- and tri-sumoylated species are only visible in the anti-SUMO immunoblot as the anti-GST antibody is not sensitive enough to detect them. The open arrowhead marks a non-specific cross-reacting band detected by the anti-GST antibody.

To determine if SUMO plays a role in Ras signaling, we knocked down SUMO by RNAi in cultured S2R+ cells, which express *Drosophila* EGFR [54], and examined the level of MAPK activation in these cells upon activation of EGFR by the secreted Spitz ligand, sSpi [55]. Anti-SUMO immunoblotting showed that the treatment with SUMO dsRNA progressively decreased the levels of both free SUMO and SUMO conjugates after 3 to 5 days of treatment (Figure 3A). The level of Ras pathway activation was assessed by immunoblotting for pMAPK, and all samples were normalized by comparison to total MAPK using an antibody that recognizes all forms of MAPK. The levels of pMAPK decreased with increasing duration of SUMO RNAi up to five days, and parallel treatment of cells with control YFP dsRNA had no effect on MAPK activation (Figure 3B). SUMO RNAi similarly impaired insulin-induced MAPK phosphorylation (Figure 3C). Insulin or sSpi-induced MEK activation was also reduced by SUMO knockdown (Figure 3D, normalized using the total MEK levels). To further dissect the role of SUMO in Ras signaling, we examined the requirement for SUMO in pathway activation by Ras^V12^, a constitutively active form of Ras1. SUMO knockdown did not affect Ras^V12^-induced MAPK activation (Figure 3E). This suggests that SUMO knock down does not impair the processes downstream of Ras1 activation, so SUMO pathway affects a step upstream of Ras1 or at the level of Ras1 to activate the pathway.

Ras1 was identified in the single-step native FLAG IP of sumoylated proteins (Table 2, Table S2), but not in the two-step denaturing purification protocol. However, the bacterial sumoylation assay suggests that it is nonetheless a direct sumoylation target. Sumoylated GST-Ras1 species were detected when the functional sumoylation pathway (Q^SUMO^) was co-expressed with GST-Ras1 in *E. coli* (Figure 3F), and absent when conjugation defective SUMO^ΔGG^ (Q^ΔGG^) was employed instead (data not shown). There is no consensus SUMO acceptor site found in the Ras1 peptide sequence. The highest probability non-consensus site, predicted by both *SUMOsp2.0* [38] and *SUMOplot™*, is lysine 104. Mutation of lysine 104 to arginine, did not dramatically compromise Ras1 sumoylation (Figure 3F), suggesting that Ras1 contains multiple non-consensus sumoylation sites. Functional sumoylation on non-consensus sites, however, has been widely observed. For example, the yeast core histones contain multiple mass-spectrometry validated non-consensus sites that can not be identified by any of the existing SUMO prediction programs [56], [57]. Furthermore, 26% of experimentally validated SUMO conjugation sites are non-consensus [58]. We found that deletion of the C-terminal 25 amino acids of Ras1 or mutations of all lysines in this region abolished Ras sumoylation (Figure 3G), whereas the C-terminal Ras1^165–189^ peptide was sumoylated to yield a pattern of bands in SDS-PAGE similar to that observed for wild type Ras1 (Figure 3G), indicating that the hypervariable C-terminal region of Ras1 contains the sites of SUMO modification.

### SUMO is required for the syncytial mitotic cycles

**Figure 4:**
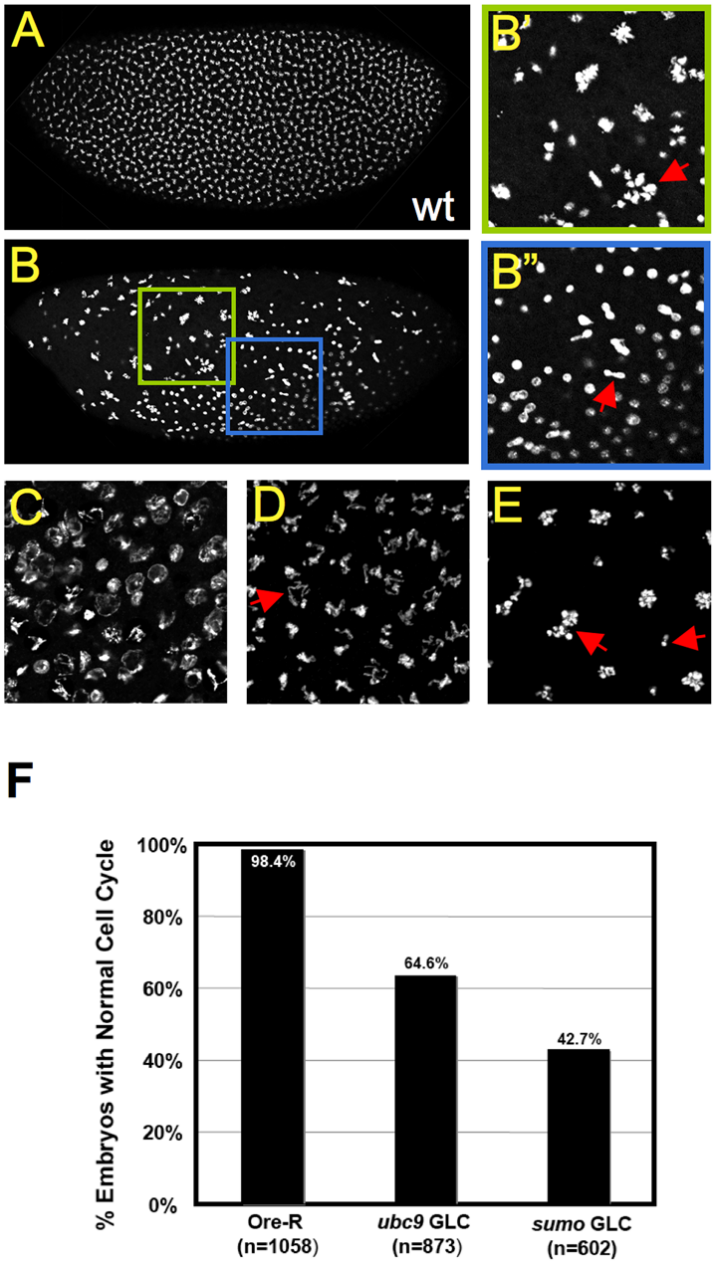
*sumo* is required for normal syncytial nuclear cycles. A) A wild type syncytial blastoderm embryo in metaphase. B–E) Representative nuclear cycle defects in DAPI stained *sumo^04493^* GLC embryos. DAPI staining revealed multiple cell cycle defects in *sumo^04493^* GLC embryos. Panel B shows a *sumo^04493^* mutant embryo, while B′ and B″ are magnified views of two regions of the embryo in B. The arrow in B′ points to an abnormally large cluster of chromosomes, indicating polyploidy, and the arrow in B″ points to a prominent chromosome bridge. C) Abnormal chromosomal organization. The arrow in D highlights a possible cohesion defect. The left arrow in E points to a cluster of hypercondensed chromosomes, and the right arrow points to chromosome fragments. F) Frequency of cell cycle defects in *sumo* and *ubc9* mutant GLC embryos.

The SUMO pathway has been shown to be required for cell cycle progression in other organisms [59]. Consistent with this, our proteomic analysis found proteins involved in cell cycle regulation to be significantly over-represented among SUMO conjugates in the early *Drosophila* embryo (Table 2, Table S6), and moreover our findings significantly expands the list of know sumoylated cell cycle regulators. To determine if the lethality caused by a reduced maternal supply of SUMO is due to cycling defects, 0- to 3-hour wild-type and *sumo^04493^* GLC embryos were stained with DAPI to visualize DNA. During the initial stage of *Drosophila* embryogenesis, 13 nuclear cleavage cycles occur rapidly and synchronously in a syncytium (Figure 4A). We observed that over 50% of the *sumo^04493^* GLC embryos exhibited a broad spectrum of nuclear cycle defects, including irregular size and distribution of nuclei, asynchronous nuclear division, abnormal interphase chromosome structure, overly condensed chromosomes, loss of sister chromatid cohesion during metaphase, polyploidy, chromosome clustering, fragmentation, and chromosome bridges (Figure 4B–E). Multiple nuclear division defects were often observed in a single embryo (Figure 4B). We also observed similar, although somewhat less penetrant, nuclear cleavage cycle defects in embryos resulting from GLC of a *ubc9* (the SUMO conjugating enzyme) hypomorphic allele [12], *semi^118^* (Figure 4F). The diverse cycling defects observed in the *sumo* and *ubc9* GLC embryos indicate broad involvement of SUMO in multiple stages of the nuclear cycle, and are consistent with our proteomic analysis showing a significant enrichment in SUMO targets with cell cycle functions.

**Figure 5:**
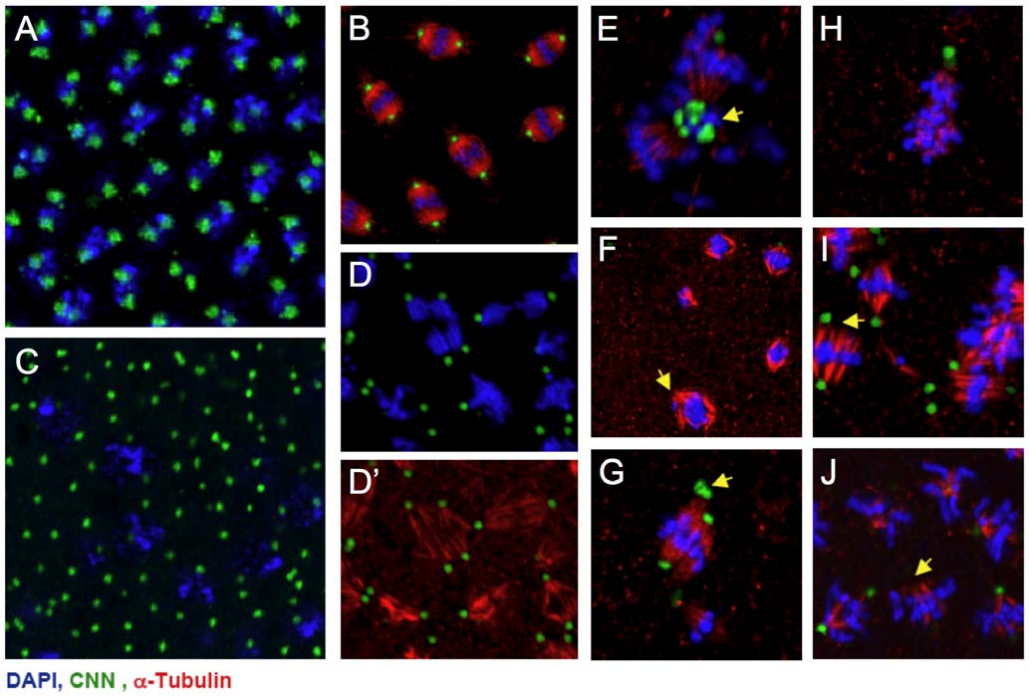
Defects in the coordination of centrosome replication and spindle attachment in *sumo* GLC embryos. A–B) Syncytial blastoderm Ore-R embryos. C) Uncoupled chromosome and centrosome replication observed in *sumo^04493^* GLC embryos. D–J) In *sumo^04493^* GLC embryos, multiple defects in metaphase spindle morphology are observed, including examples of anastral (arrow in F), monoastral (H), multipolar (E, G, arrows), as well as unfocused mitotic spindles (D′ and arrows in I, J). The embryos were stained for DAPI (blue), centrosomin (CNN, green), and α-Tubulin (red).

The mitotic cycle defects in *sumo* mutant GLC embryos were further characterized through visualization of centrosomes and microtubules. Correct spatial organization and synchronous nuclear division of the early embryos requires a high degree of coordination between centrosome duplication, microtubule dynamics, and changes in nuclear structure. Abnormalities, such as asynchronous division, irregular nuclear spacing, and polyploidy, observed in *sumo^04493^* GLC embryos, suggest an uncoupling of these events. In wild type syncytial blastoderm embryos, each set of chromosomes is associated with a pair of centrosomes (Figure 5A, B). *sumo^04493^* embryos often contain a reduced number of nuclei in relation to the centrosome pairs (Figure 5C), a common mitotic defect in *Drosophila* [60]–[62].

Defects in mitotic spindle organization and attachment to centrosomes and chromosomes were also observed in *sumo* GLC embryos (Figure 5D–J). Monoastral, anastral, and multipolar spindles, as well as unfocused broad-based spindles were documented. These results suggest that sumoylation is important in coordinating multiple events of mitosis, such as centrosome replication, centrosome-spindle association, and spindle-chromosome attachment. The broad spectrum of mitotic defects seen in the *sumo* GLC embryos is consistent with the defective mitotic spindle assembly induced by SUMO RNAi in *Drosophila* S2 cells previously observed [63], as well as with our fly SUMO-ome, which also suggests a broad role for SUMO in mitosis.

### SUMO is required for cell cycle progression in S2 cells and larval tissue

**Figure 6:**
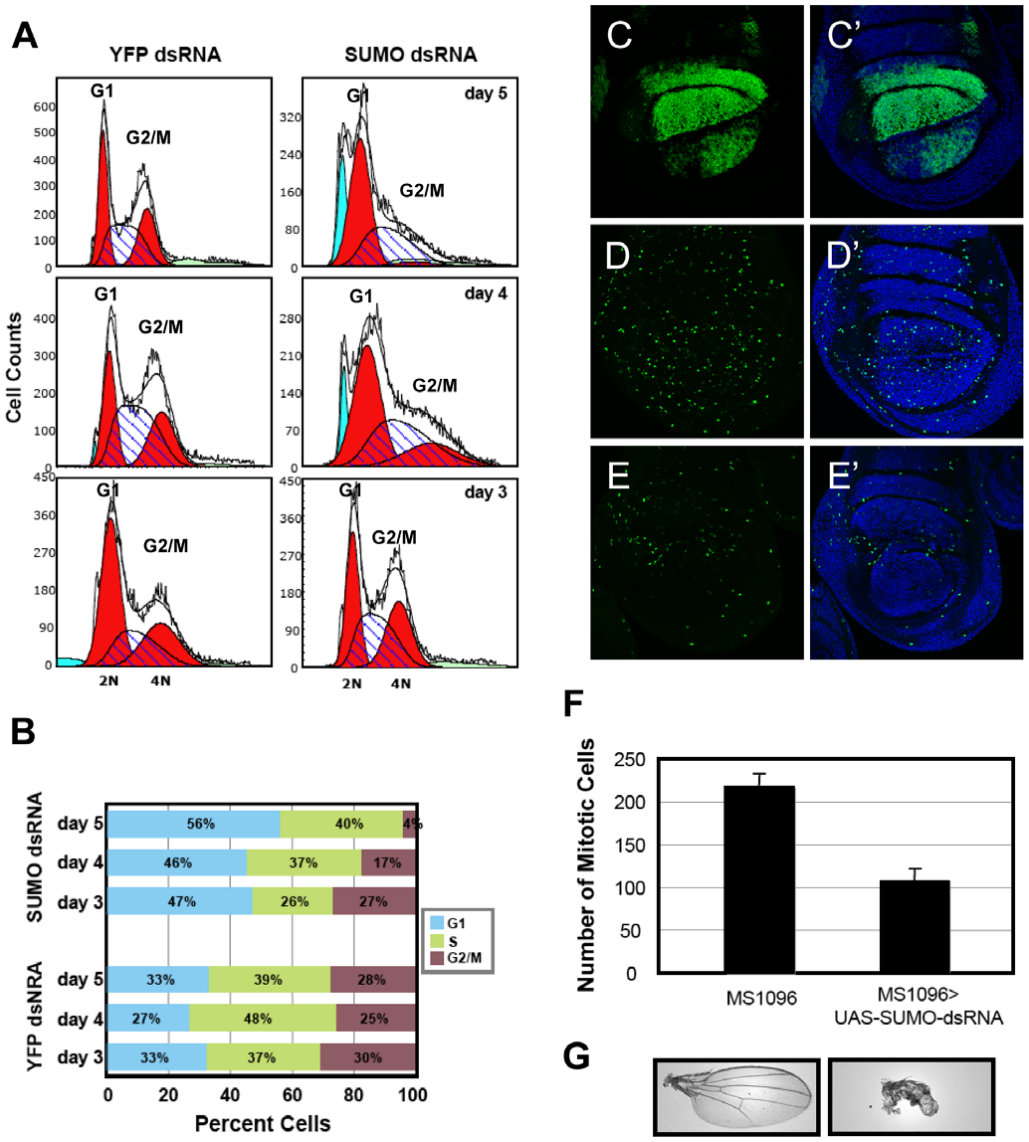
SUMO is required for cell cycle progression in cultured *Drosophila* cells and in larval tissues. A) FACS analysis of DNA content in S2 cells treated with dsRNA against SUMO or YFP for 3, 4, or 5 days. The overall distribution (unfilled curve) has been fit to show the G1 (2N) and G2/M (4N) cells (red-filled curves), the S phase cells (hatched curve), cells with less than 2N DNA content (blue-filled curves), and cells with more than 4N DNA content (green-filled curves). B) The percentages of YFP or SUMO dsRNA treated cells in G1, S, and G2/M phases. C–G) SUMO is required for mitosis in wing imaginal discs. C) This *MS1096>laminGFP* wing disc shows the domain of *MS1096-Gal4* expression. Mitotic cells were marked by pHH3 staining in *MS1096* (D), or *MS1096>sumoRNAi* (E) third instar larval wing discs (all discs are oriented with dorsal on the top and anterior on the right). C′, D′, and E′ show DAPI overlay of the images on the left. F) The numbers of pHH3 positive cells in multiple discs were counted and averaged (standard errors are indicated). G) Adult wings from the *MS1096* (left) or *MS1096>sumoRNAi* (right) flies.

The syncytial nuclear cleavage cycles are non-canonical mitotic cycles lacking the gap phases as well as cytokinesis. To determine if sumoylation is essential for cells undergoing a canonical G1-S-G2-M cell cycle, we investigated the requirement for SUMO in cell cycle progression of cultured cells and larval wing imaginal discs. The DNA content of S2 cells was measured by flow cytometry to assess cell cycle stage following three to five days of SUMO RNAi. The fraction of G2/M phase cells gradually diminished, suggesting G1/S arrest as a result of sustained low levels of SUMO (Figure 6A, B).

Imaginal discs undergo rapid proliferation during larval development. We knocked down SUMO in larval tissue by RNAi using Gal4 to drive the expression of a *sumo* hairpin RNA. The effectiveness of the RNAi was demonstrated by anti-SUMO staining of discs containing clones of cells that express *sumo* hairpin RNA (Figure S5). To examine the effects of SUMO knockdown on the wing imaginal disc cell cycle, we employed the *MS1096-Gal4* driver, which directs a high level of expression in the dorsal compartment and a lower level of expression in the ventral compartment of the third instar wing pouch (Figure 6C, C′). The wing discs were stained with an antibody against phosphohistone H3 (pHH3) to mark the mitotic cells (Figure 6D–E′). *sumo* RNAi resulted in a marked reduction of pHH3 positive cells throughout the wing pouch (Figure 6E, E′, F). The proliferation defect was especially pronounced in the dorsal compartment where the Gal4 driver is most active (Figure 6E, E′), and was also manifested in adult wings, which were greatly reduced in size and misshapen (Figure 6G). A similar requirement for SUMO in cell proliferation was also observed in eye discs when *ey-Gal4* was used to drive the expression of *sumo* hairpin RNA (data not shown). Thus, SUMO is required for both the atypical mitotic cycle that occurs in nuclear cleavage stage embryos as well as the canonical cell cycles that occur in cultured cells and larval imaginal discs.

### Dynamic localization of SUMO during mitosis

**Figure 7:**
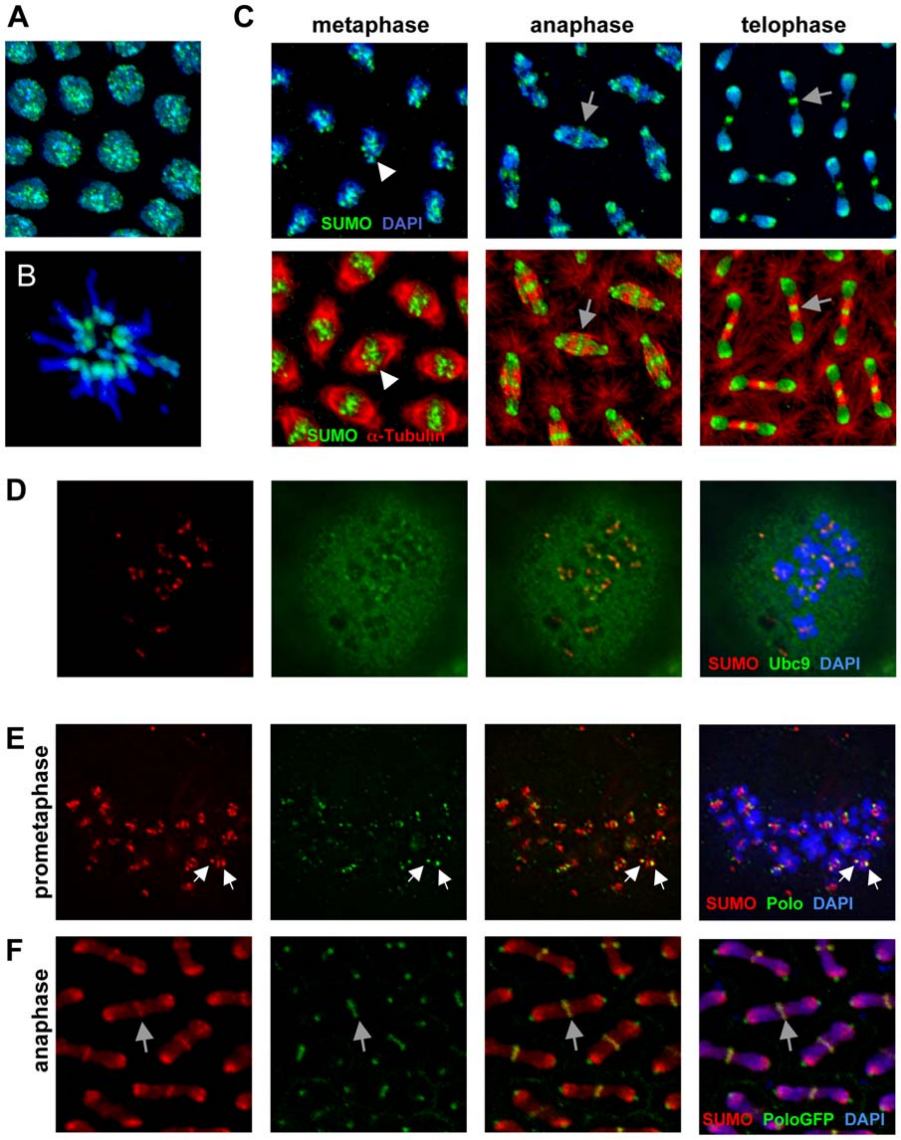
SUMO localization throughout the mitotic cycle. A) Interphase nuclei of syncytial blastoderm stage Oregon-R embryo stained with SUMO antibodies (green) and DAPI (blue). B) Polar body stained with SUMO antibodies (green) and DAPI (blue) showing SUMO localization to the pericentromeric regions of the polar body chromosomes. C) Metaphase, anaphase, and telophase nuclei of syncytial blastoderm embryo stained with SUMO antibodies (green), α-tubulin antibodies (red), and DAPI (blue). Top panels show the SUMO/DAPI merge, while bottom panels show the SUMO/α-tubulin merge. White arrowheads in left panels point to pericentromeric SUMO in one of the nuclei. Gray arrows in the center and right panels point to spindle midzone SUMO. D) HA-Ubc9-expressing S2 cells [23] arrested at prometaphase with 25 µM colchicine and stained with SUMO antibodies (red), HA antibodies (green, revealing localization of HA-Ubc9), and DAPI (blue). Ubc9 is diffusely localized but also exhibits intense puncta that colocalize with SUMO near the kinetochores. E) S2 cell arrested at prometaphase with colchicine and stained with DAPI (blue), and antibodies against SUMO (red) and Polo (green). SUMO and Polo partially colocalize at the outer kinetochore regions (white arrows point to the kinetochores of one pair of sister chromatids). F) Syncyticial embryo expressing Polo-GFP (green; Polo-GFP flies were obtained from Dr. Claudio Sunkel) was stained with DAPI (blue) and antibodies against SUMO (red). Polo and SUMO colocalize to the midbody (indicated for one nucleus by the gray arrow).

Our immunofluorescence studies on *sumo* GLC embryos clearly indicate that SUMO plays diverse roles at various stages of the mitotic cycle. To gain further insight into the role of SUMO in the cell cycle, we systematically documented the localization of SUMO through stages of the nuclear cleavage cycle (Figures 7A, C). Previous studies have shown that SUMO assumes a predominantly nuclear distribution in the early embryo at interphase [16]. This observation was confirmed in this study. During interphase and prophase, SUMO is distributed throughout the nucleus, but is concentrated in puncta of unknown structure (Figure 7A). As the embryonic nuclei progress to metaphase, SUMO associates with the condensing chromosomes and appears to be concentrated in regions around the centromeres, as marked by the points of closest association between the spindle and the chromosomes (Figure 7C, left panels). This pericentromeric localization is also consistent with the central ring of anti-SUMO staining observed in the polar body chromosomes (Figure 7B), and with previous observations of SUMO association with the chromocenter of the polytene chromosome [16], [22].

The association of SUMO with the pericentromeric regions of the chromosomes persists during anaphase and telophase (Figure 7C, center and right panels). In addition, SUMO also localizes to the spindle midbodies, which are clearly framed by the midzone spindles (the spindles between the segregating sister chromatids), as they form during anaphase (Figure 7C, center panels). Localization of SUMO to the midbody is even more apparent during telophase (Figure 7C, right panels). The association of SUMO with the chromosomes and midbody was also observed in S2 cells (Figure 7E and data not shown). The association of SUMO with the chromosomes throughout mitosis and its localization to the midbodies during anaphase and telophase is consistent with our observation that SUMO plays critical roles during multiple stages of the mitotic cycle. These findings are also consistent with a report that SUMO-2/3 in HeLa cells associates with the mitotic chromosome, while SUMO-1 associates with the spindle midzone [64]. Apparently, the multiple mitotic functions of SUMO carried out by the single SUMO isoform in *Drosophila* have been divided among multiple SUMO isoforms in vertebrates.

The localization of SUMO during mitosis can be observed in more detail in cultured cells. Cells arrested at prometaphase by colchicine treatment display SUMO localization on condensed chromosomes at the outer kinetochore and inner centromeric regions (ICR) (Figure 7E). Like SUMO, Ubc9 assumes a predominantly nuclear distribution during interphase (data not shown). During mitosis, Ubc9 staining exhibited a largely diffuse distribution along with concentrated foci that overlapped the domain of SUMO localization at the centromeric regions and kinetochores, suggesting that active sumoylation is taking place at those locations (Figure 7D).

Polo, one of the SUMO targets identified in our proteomic screen and validated using the bacterial sumoylation system (Table 2, Figure S4C), is the only *Drosophila* Polo-like kinase family protein. Polo is involved in multiple stages of cell cycle regulation, localizes to the outer kinetochore early in mitosis, and subsequently relocalizes to the midbodies late in mitosis [65]. Given these similarities between the functions of SUMO and Polo, we decided to compare directly the localization of SUMO and Polo during the mitotic cycle. During interphase, Polo and SUMO occupy distinct cellular compartments, being cytoplasmic and nuclear, respectively (data not shown). During prometaphase and metaphase, partial overlap between SUMO and Polo is observed at the outer kinetochore (Figure 7E, and data not shown). At later stages of mitosis (anaphase and later), Polo is localized to the midbody, and again exhibits incomplete overlap with SUMO (Figure 7F). This partial co-localization during multiple phases of mitosis suggests that Polo could be one of the SUMO substrates at the kinetochores. However, it also indicates that there are likely additional SUMO substrates at the kinetochores and inner centromeric region, and that not all kinetochore-associated Polo is sumoylated.

## Discussion

The SUMO conjugation pathway is highly conserved in eukaryotic evolution, and plays many key regulatory roles. *Drosophila* embryos contain high levels of maternally supplied SUMO, indicating that sumoylation may be especially important in early *Drosophila* embryogenesis. Accordingly, reduced maternal expression of SUMO has pleiotropic effects in oogenesis and embryogenesis. Our proteomic, genetic, and cell culture analyses converge to support roles for protein sumoylation in Ras signaling, mitotic progression, and embryonic pattern formation.

### SUMO and Ras signaling

The Ras signaling cascade is activated by a variety of RTKs including EGFR, and controls cell proliferation and differentiation as well as a large number of developmental patterning processes, such as patterning of the eggshell [66]–[68]. Activation of EGFR in the dorsal follicle cells during oogenesis leads to the sequential activation of Ras, Raf, MEK, and MAPK, and results in the upregulation of RTK target genes [69]. Complex positive and inhibitory feedback loops ultimately result in the specification of the dorsal follicle cells, which later secrete the dorsal eggshell, including the dorsal appendages [52], [70].

Previous genetic screens for mutations that enhance the eggshell ventralization phenotype of a weak hypomorphic *Ras1* allele suggested a role for SUMO in the Ras pathway downstream of EGFR activation [53]. In our analysis of the recessive *sumo* mutant phenotype, we observed fused or single dorsal appendages, indicative of eggshell ventralization and consistent with the attenuation of EGFR signaling. Since the eggs under study resulted from *sumo* GLCs, the observed eggshell defect could reflect a function for SUMO upstream of EGFR in the production or secretion by the germ line of EGFR ligands. However, since *sumo* mutant clones are also present in the follicle cells of the GLC egg chambers, the eggshell ventralization phenotype we observe is also consistent with a role for SUMO downstream of EGFR activation in the follicle cells. Interestingly, sumoylation pathway proteins in *C. elegans* were also shown to interact with the Ras signaling pathway [71]. Our cell culture experiments support a role for protein sumoylation in Ras signaling that is downstream of EGFR and upstream of, or parallel to, Ras activation. SUMO may directly modulate Ras1 function since Ras1 was found in our proteomic analysis and confirmed as a sumoylation substrate in our bacterial sumoylation assay.

### SUMO and cell cycle progression

Sumoylation is implicated in cell cycle regulation in many organisms [59]. In this study, we observed diverse nuclear cleavage defects in *sumo* GLC embryos suggestive of multiple roles for SUMO in coordinating the chromosome cycle. The phenotypes, including chromosome hypercondensation, aberrant segregation, and polyploidy, are reminiscent of the defects observed in Ubc9-deficient mouse embryos and *Drosophila* embryos mutant for *pias*, a possible SUMO ligase [13], [72], indicating conservation of SUMO cell cycle functions in metazoan evolution. We also demonstrated a requirement for SUMO in cell cycle progression in cultured cells and in larval imaginal discs by RNAi-mediated SUMO knockdown. While the cell proliferation defect in SUMO mutant wing discs could result from a requirement for SUMO for the function of many of the same cell cycle proteins found in our proteomic screen of early embryos, it could also reflect a role for SUMO in the function of Vg, a previously identified wing disc sumoylation target [19] known to be required for wing growth [73]–[75].

In agreement with the diverse cell cycle defects in *sumo* mutant embryos and other tissues, a spectrum of cell cycle regulators involved in multiple stages of the cell cycle were identified in our SUMO proteomic screens (Table 2). For example, the failure of cultured cells to progress to G2/M could reflect a role for SUMO in DNA replication, which is consistent with our finding that PCNA, RFC2, Topoisomerase I, and Topoisomerase II are all targets of sumoylation (Table 2 and Figure 1C). A role for SUMO in the function of Polo kinase could further explain some of the observed cell cycle defects since Polo has multiple roles in the cell cycle [65], [76]. Other sumoylation targets identified in our screen, including PP2A, Arp3, Cofilin (Twinstar), Mago Nashi, and Profilin, are also consistent with multiple roles of SUMO in mitosis.

The requirement for SUMO throughout mitosis is further supported by its dynamic, mitotic stage-dependent, localization. At prometaphase and metaphase, sumoylated proteins are concentrated at the kinetochores and ICR, partially co-localizing with Polo. Ubc9 co-localized with SUMO at the kinetochore-centromeric regions during mitosis, suggesting that active sumoylation is taking place at those locations. It is likely that many kinetochore and centromere localized proteins are targeted by SUMO, and cycles of sumoylation and de-sumoylation may help to propel unidirectional mitotic progression.

While a number of studies have connected sumoylation to centromere and kinetochore functions [59], spindle midbody localization of SUMO has not been widely reported [64], [77]. The midbody is a structure derived from the spindle midzone that contains proteins indispensable for cytokinesis [78]. SUMO association with the midbody, which we have observed in both syncytial embryos and cultured cells beginning with anaphase and extending through cytokinesis, therefore argues for a role of sumoylation in the completion of cell division. The midbody proteome has been dissected recently in mammalian cells, revealing a large collection of proteins, including membrane associated proteins, microtubule associated proteins, and kinases [78]. Homologs of a number of these proteins, such as Arp3, Cofilin (Twinstar), Mago Nashi, Polo, PP2A, and Profilin, were all identified in our *Drosophila* SUMO proteomic screens (Table 2), reinforcing the notion that SUMO is involved in midbody function.

Cytokinesis does not occur in nuclear cleavage stage embryos. However, the midbody has an important role in maintaining the separation of telophase sister nuclei [79], a process that could be related to the formation of pseudocleavage furrows at the end of each nuclear cleavage cycle. Disruption of midbody function in SUMO deficient embryos may therefore account for some of the mitotic defects we observe in the syncytial embryo, including polyploidy.

### SUMO and embryonic patterning

We observe diverse patterning defects among the *sumo* GLC embryos that developed a cuticle. In accordance with this observation, three absolutely critical patterning proteins, Dorsal, Bicoid, and Hunchback, are among the sumoylated proteins we detected in early embryo extracts (Table 2). Previous studies have shown that sumoylation of Dorsal potentiates its activity during the immune response perhaps by making it a more potent transcriptional activator [18]. While an earlier study showed that the loss of Ubc9 results in a *hunchback*-like anterior patterning phenotype and defective nuclear transport of Bicoid [12], our study is the first to show that Hunchback, and its activator Bicoid, are direct SUMO conjugation targets. Thus, it is possible that sumoylation of these transcription factors plays a direct role in anterior patterning.

Posterior patterning and germ line specification depend upon the posterior localization of the *oskar* transcript. We identified several *oskar* mRNP components, including Mago Nashi, Tsunagi, Cup, Hrb27C, and Smaug, as sumoylation targets (Table 2 and 3), which have essential roles in the regulation of *oskar* mRNA localization and translation [80]. This interesting and novel finding suggests a role of SUMO in regulating the functions of maternal mRNA by modifying components of *oskar* mRNP, and therefore could explain some of the pleiotropic defects observed in the embryonic patterning of embryos resulting from *sumo* mutant GLCs.

The *oskar* mRNP is one of several instances in which multiple members of the same complex appear to be direct targets of sumoylation. For example, our screen turned up several members of the multi-aminoacyl-tRNA synthetase complex, as well as multiple ribosomal proteins (Table 3). Screens for sumoylation targets in *S. cerevisiae* have similarly detected multiple sumoylation targets in the same complex [26], [29]. This suggests that oligomeric protein complexes can be targeted as a whole for sumoylation and/or that sumoylation may have a general role in stabilizing protein complexes.

In contrast to previous studies in yeast and mammalian cell culture [26], [28], [29], [32]–[35], relatively few transcription factors were identified in our study. This difference in fact accurately reflects the unique metabolic state of the pre-cellularization embryo. During the first two hours of *Drosophila* embryonic development, rapid nuclear divisions depend upon a complex dowry of maternally supplied proteins, as transcription of the zygotic genome has not yet begun. Instead, the proper localization and accurately regulated translation of maternally supplied mRNAs is essential for establishing the system of positional information that will later direct the spatially regulated transcription of the zygotic genome [81]. Thus, the relatively small and selective group of sumoylated transcription factors, along with the large number of factors that control mRNA translation and localization found in our screen, is consistent with regulatory roles for SUMO in this critically important stage of fly development.

In conclusion, our genetic, cellular, and proteomic studies of sumoylation suggest mechanisms for known biological roles of the SUMO pathway and also uncover novel connections between sumoylation, signal transduction, the cell cycle, and development. Furthermore, our SUMO conjugated proteome should serve as a rich resource for those studying the roles of sumoylation in metazoan development.

## Materials and Methods

### Plasmid construction

Sequences encoding the (His)_6_- and FLAG-tags (HHHHHHDYKDDDDK) were added to the 5′ end of the *sumo* coding region by PCR, using primers containing the sequences corresponding to those tags as well as *Not*I and *Xba*I restriction sites (primer sequences are given in supporting document S1). The resulting PCR product was digested and ligated into the pUASp vector [82] to produce pUASp-H_6_Flag-SUMO.

To construct the plasmid termed “Quartet” or “Q”, which expresses the *Drosophila* SUMO pathway in a single vector, components of the pathway were first cloned into two Duet vectors (Novagen), then combined into a single vector. Briefly, the mature form of SUMO, SUMO^GG^ (last 2 amino acids omitted), and *ubc9* were amplified from cDNAs by PCR, and cloned into pRSF-Duet-1 MCS1 at the *Eco*RI/*Not*I sites, and MCS2 at the *Nde*I/*Xho*I sites, respectively, to generate pRSF-SUMO^GG^-Ubc9. Similarly, *sae2* and *sae1* were cloned into the MCS1 and MCS2 of the pCDF-Duet-1 to create pCDF-SAE2-SAE1. Subsequently, the pCDF-SAE2-SAE1 was digested with *Pfo*I, filled in with Klenow, and then cut at the *Age*I site, and the *sae2-sae1* fragment was then introduced by ligation into the pRSF-SUMO-Ubc9 vector, which has been digested with *Bsu*36I, blunted using Klenow, and then digested with *Age*I, to obtain, Q^SUMO^ (pRSF-SUMO^GG^-Ubc9-SAE2-SAE1). The control vector, Q^ΔGG^ (pRSF-SUMO^ΔGG^-Ubc9-SAE2-SAE1), which expresses a conjugation defective form of SUMO, SUMO^ΔGG^, in place of SUMO^GG^, was constructed using the same strategy. The ORFs of *sumo* and *sae2* were cloned in frame with an N-terminal (His)_6_ tag, and *ubc9* and *sae1* were cloned in frame with a C-terminal S-tag.

The pGEX-loxP plasmid was generated by inserting a sequence containing the *loxP* recombination site and bacterial promoter, for Cre recombination and an antibiotic selection gene, respectively (sequence information is available in The Creator Cloning System Manual, Clontech), into pGEX-4T-1 (Amersham) at the *Eco*RI and *Xho*I sites. This vector serves as an acceptor vector for generating an in-frame amino terminal GST fusion with open reading frames (ORFs) that have been introduced into a donor vector. The donor pDNR-Dual vectors for *PCNA* (BS06345), *RfC2* (BS06321), *CtBP* (BS10020), *HP1* (BS03857), and *Ras1* (BS04665) were purchased from the Drosophila Genomics Resource Center (DGRC). The ORFs of *14-3-3ζ, squid*, *polo*, and *eIF4E* were amplified from cDNA clones (RH61958, LD29474, BO04660, and RE36735 from the DGRC) by PCR, and introduced into pDNR-Dual (Clontech) at the *Hind*III and *Sal*I sites utilizing the In-Fusion PCR Cloning Kit (Clontech). The ORFs were then transferred from the pDNR vectors into the acceptor, pGEX-loxP vector, by Cre recombination (Clontech). The *eIF4E* and *Ras1* ORFs were also recombined into the S2 cell expression acceptor vector, pMK33FlagHis-BD (obtained from Dr. Mark Stapleton).

The Ras^K104R^ point mutation was generated by PCR-based site-directed mutagenesis of pDNR-Dual-Ras1. The Ras^1–164^, Ras^7KR^, and Ras^165–189^ were cloned into pDNR-Dual vector by insertion of PCR products (see supporting document S1 for primer sequences). The sequences encoding these Ras variants were then recombined into the pGEX-loxP vector. The Ras^V12^ point mutation was created by PCR-based mutagenesis of the pMK33FlagHis-Ras1 vector. All plasmids generated in this study were sequenced to verify the presence of the correct inserts and sequences (UCLA Genotyping and Sequencing Core).

### Two-step purification of SUMO conjugates

Wild type Oregon-R embryos or embryos expressing tagged SUMO, were collected at 25°C over a three-hour period, washed, incubated at 37°C for an additional 45 minutes, then immediately frozen under liquid nitrogen. Five grams of frozen embryos were ground to power under liquid nitrogen and suspended in 20 ml of Urea Binding buffer **A** (100 mM NaH_2_PO_4_, 10 mM Tris, pH 8.0, 8 M urea, 5 mM imidazole, and 1% CHAPS), which was freshly supplemented with 40 mM N-ethylmaleimide (NEM, Sigma) and one Mini Complete Protease Inhibitor Cocktail Tablet (Roche). The suspension was further lysed with a French Press, and the lysate was centrifuged at 32,000×g at 4°C for 20 min. The supernatant was filtered using Miracloth (CBC) to remove lipid clumps, and then mixed with 1 ml of buffer A equilibrated Ni-NTA beads (QIAGEN) at 22°C for one hour and 30 min in an Econo-column (BioRad). After removal of the unbound material by gravity flow, the Ni-NTA beads were washed twice with total of 40 ml of Wash buffer **B** (100 mM NaH_2_PO_4_, 10 mM Tris, pH 8.0, 8 M urea, 5 mM imidazole, and 1 mM PMSF), and eluted 4 times with 1 ml of Elution buffer **C** (100 mM NaH_2_PO_4_, 10 mM Tris, pH 8.0, 8 M urea, 20 mM EDTA, and 400 mM imidazole).

The eluted proteins were immediately dialyzed against three liters of TBS (50 mM Tris, pH 7.4, 300 mM NaCl, 1 mM EDTA, and 5% glycerol) over 2 hours at 4°C. The dialyzed sample was further diluted five-fold with cold TBST buffer (TBS plus 1% Triton X-100, and supplemented with 20 mM NEM and protease inhibitor cocktail), and incubated with 100 µl of anti-FLAG agarose (Sigma) at 4°C overnight. The next day, the beads were separated from unbound proteins by centrifugation, washed four times with TBST, and transferred to a spin collection column (Zymo) to remove TBST. The proteins were eluted from the beads with NuPAGE LDS loading buffer (Invitrogen) at 70°C for 15 min, and a portion of the eluted proteins were later separated by SDS-PAGE and analyzed by Western blot or in-gel trypsin digest followed by LC-MS/MS analysis of the tryptic peptides.

### Protein identification by LC-MS/MS

The sliced SDS-PAGE gels were digested with sequencing-grade trypsin (Promega). The digested peptides were extracted from the gel slices using 50% acetonitrile/0.1% trifluoroacetic acid (TFA) in water. The extracts were dried down, resuspended in 0.1% formic acid/water, and LC-MS/MS of the peptide mixtures was performed on an Applied Biosystems QSTAR XL (ESI-QqTOF) mass spectrometer coupled with an LC Packings nanoflow HPLC system, through a nanoelectrospray ionization source (Protana). A homemade trap column (150 µm×2 mm) and nano-column (75 µm×150 mm) packed with Jupiter Proteo C12 resin (particle size 4 µm, Phenomenex) were employed for the nano-flow HPLC peptide separation using an 80-minute gradient. Product ion (MS/MS) spectra of the peptides separated by HPLC were recorded and then submitted to the Mascot database search engine (Matrix Science) for protein identification. Positive protein identification was based on standard Mascot scoring criteria (*p*<0.05) for statistical analysis of the LC-MS/MS data. The peptide assignments in the Mascot database search results were then manually inspected for validation.

### The bacterial sumoylation assay

A vector encoding a candidate SUMO conjugation target fused to GST was co-transformed into BL21 cells (Novagen) with either the Q^SUMO^ or the control Q^ΔGG^ expression vectors. Several transformed bacterial colonies, selected for kanamycin and ampicillin resistance, were picked to inoculate 0.5 ml cultures of non-inducing medium, and shaken at 37°C overnight. This culture was then used to inoculate 0.5 ml culture in auto-inducing medium overnight at 25°C to induce expression of all five proteins. The auto-induction was carried out as described by F. William Studier [83]. These small-scale cultures were compared for levels of expression of the GST fusion protein by anti-GST Western blot. The culture with the highest expression was then scaled up to a 50 mL culture. The bacteria grown to saturation (O.D._600_ of 12–18) were collected by centrifugation, and the GST-fusion protein was purified with glutathione beads according to the manufacturer's protocol (Amersham). The eluted proteins were subsequently resolved by SDS-PAGE, and analyzed by Sypro Ruby protein staining and immunoblotting.

### Fly stains and crosses

Flies were maintained on standard medium at 25°C. The pUASp-H_6_Flag-SUMO vector was introduced into *w^1118^* flies by embryo injection (Model System Genomics of Duke University). Multiple lines with insertions into the X, second, or third chromosomes were recovered. The *MatGal4* driver, which encodes Gal4-VP16 under the control of a maternally active *α4-Tubulin* promoter was generously provided by Dr. Daniel St. Johnston.

The *sumo* mutant fly stocks used in this study are *P[ry^[+t7.2]^ = PZ], smt3^04493^/CyO; ry^506^* (referred to as *sumo^04493^* in this study; obtained from Bloomington Stock Center) and *Df^1^w^67c23^, y^1^; P[lacW]smt3^k01211^/CyO* (referred to as *sumo^k01211^*; obtained from Dr. Jon Schnorr). The original *CyO* balancer of the *sumo* mutant lines was replaced with a GFP expressing *CyO* balancer (*CyO, ActGFP*) to allow homozygous SUMO mutant flies (which lack GFP) to be distinguished from their heterozygous siblings. The fly strain containing a second chromosome insertion that expresses a hairpin RNA against the SUMO gene under UAS control (*UAS-sumoRNAi*) was obtained from the Vienna *Drosophila* RNAi Center. Since the *UAS-sumoRNAi* flies are homozygous sterile, we balanced the line with *CyO*, *ActGFP* to allow the *UAS-sumoRNAi* carrying flies to be distinguished.

An FRT site (FRT40A) was introduced by recombination onto the chromosome arms carrying the mutant *sumo* alleles. The FRT40A line of mutant *ubc9*, *semi^118^*, was a obtained from Dr. Sochi Tanda [12]. The standard dominant female sterile FLP/FRT protocols were followed to generate germ line clones of *sumo P*-element alleles or *semi^118^* [49].

### Scanning electron microscopy and cuticle preparation of embryos

Fly embryos were attached to metal mounts as uncoated samples using fingernail polish. Images were digitally acquired using a Hitachi S-2460N Scanning Electron Microscope at a ‘high pressure’ setting of 30 Pa using a Robinson detector. To prepare embryo cuticles, dechorionated and devitellinized embryos were mounted on slides in Hoyer's mounting medium [84], and imaged with dark field optics on a Zeiss Axioskop microscope.

### Immunofluorescence and Western blotting

Primary antibodies used for immunofluorescence were rabbit anti-SUMO [23], mouse anti-α-tubulin (Sigma), rabbit anti-CNN [85], rabbit anti-pHH3 (pSer10, Sigma), mouse anti-Polo (a gift of Dr. Claudio Sunkel), and mouse anti-HA (Sigma). Secondary antibodies used were goat anti-rabbit or goat anti-mouse antibodies conjugated with Alexa Fluor 488 or Alexa Fluor 568 (Molecular Probes). DNA was stained with 1 µg/ml 4′,6-diamidino-2-phenylindole (DAPI). Confocal images of the embryos and imaginal discs were obtained on a Leica one-photon confocal laser scanning microscope (Leica Microsystems, Heidelberg). The S2 cells were visualized on a Deltavision Spectris deconvolution microscopy system (Applied Precision), and the images were deconvolved using Applied Precision software.

Antibodies used for immunoblotting were rabbit anti-ERK (Sigma), mouse anti-dpERK (Sigma), rabbit anti-MEK (Cell Signaling), rabbit anti-pMEK (Cell Signaling), mouse anti-FLAG (Sigma), rabbit anti-SUMO [23], rabbit anti-GST (Abcam), and mouse anti-poly-His (Sigma). Signal detection was achieved with secondary antibody conjugated with horseradish peroxidase (HRP) (CalBiochem) and SuperSignal West Pico substrates (Pierce).

### Cell culture, RNA interference, and cell cycle analysis

*Drosophila* cultured cells were maintained at 24°C in Schneider's insect medium (Gibco) supplemented with 10% fetal bovine serum (JHR) and antibiotics (Invitrogen). PCR products with T7 promoters on both ends (primer information is given in supporting document S1) were used as templates for *in vitro* transcription to make dsRNAs using the Megascript RNAi kit (Ambion). The dsRNA was introduced into the cultured cells as described [86]. To establish stable cell lines expressing FLAG-(His)_6_-Ras^V12^ or FLAG-(His)_6_-eIF4E under inducible condition, the pMK33FlagHis-Ras^V12^ or pMK33FlagHis-eIF4E plasmid was transfected into S2 cells using Effectene (QIAGEN). The transfected cells were then selected with hygromycin until stable cell lines were established. For the flow cytometry, cells were suspended in Propidium Iodide DNA staining buffer [87], and analyzed on a Becton Dickinson FACScan Analytic Flow Cytometer at the UCLA Flow Cytometry Core Facility.

## Supporting Information

Table S1 SUMO conjugates identified in two-step purification under denaturing conditions
(0.05 MB XLS)

Table S2 Proteins identified in one-step purification under native conditions
(0.06 MB XLS)

Table S3 Comparison of proteins found in one-step purification from heat shocked and non-heat shocked embryos
(0.24 MB XLS)

Table S4 GO categories of SUMO conjugates identified in two-step purification - biological processes ontology
(0.04 MB XLS)

Table S5 GO categories of SUMO conjugates identified in two-step purification - cellular component ontology
(0.03 MB XLS)

Table S6 Comparison on the levels of enrichment in Biological Processes of the 2h AEL embryos proteome to that of the SUMO conjugates identified in two-step purification
(0.03 MB XLS)

Document S1 Supplementary methods and figure legends
(0.09 MB DOC)

Figure S1 Detection of native and tagged SUMO in an anti-SUMO immunoblot
(0.35 MB TIF)

Figure S2 FLAG-IP of embryos expressing tagged-SUMO or control embryos
(0.88 MB TIF)

Figure S3 *sumo* P-element mutant expresses reduced levels of *sumo* mRNA
(0.22 MB TIF)

Figure S4 Bacterial sumoylation assays on HP1, squid, and Polo
(0.50 MB TIF)

Figure S5 Efficient knockdown of SUMO with dsRNA in larval tissues using a Gal4/UAS system
(3.20 MB TIF)
