# Supplementary material for: Genetic and Proteomic Evidence for Roles of Drosophila SUMO in Cell Cycle Control, Ras Signaling, and Early Pattern Formation
Source: PLoS One. 2009 Jun 16;4(6):e5905. doi: 10.1371/journal.pone.0005905 (PMC2692000; doi:10.1371/journal.pone.0005905)
Supplement: Document S1 — Supplementary methods and figure legends (0.09 MB DOC) [file pone.0005905.s007.doc]

**SUPPLEMENTARY METHODS**

**RT-PCR analysis of *sumo* transcript levels**

Homozygous offspring from *sumo^04493^/CyO, ActGFP* parents survived to the first larval instar, and could be distinguished from the heterozygous larvae by the lack of GFP fluorescence. Fifty newly hatched wild type Oregon-R (Ore-R) or *sumo^04493^* homozygous first instar larvae were collected, from which total RNA was isolated using the Trizol reagent according to the manufacturers instructions (Invitrogen). Any residual DNA was degraded with RNase-free DNase (Promega). The reverse transcription, and subsequent PCR reactions, were performed using the Access RT-PCR kit (Promega).

**Clonal expression of SUMO dsRNA in flies**

Clonal expression of double-stranded RNA (dsRNA) against SUMO was achieved by crossing *hs-flipase*; *vgQ-lacZ*; *Actin-FRT-CD2-FRT-Gal4/TM6B* females with *UAS-sumoRNAi/CyO, ActGFP* males. Following two-days of egg laying, the progeny were allowed to develop for an additional 48 hours, and then subjected a 40-minute heat shock at 35°C. After 2 or 3 days, late-third-instar larvae lacking TM6B and GFP fluorescence were selected for dissection and staining.

## Antibodies

The primary antibodies used in the supplemental data were rabbit anti-SUMO (1:1,000) and mouse anti-CD2 monoclonal (Serotec; 1:500). Secondary antibodies used were goat anti-rabbit and goat anti-mouse antibodies conjugated with Alexa Fluor 488 and Alexa Fluor 568, respectively (Molecular Probes). DNA was stained with 1 μg/ml 4',6-diamidino-2-phenylindole (DAPI).

**One-step purification of (H)_6_-FLAG-tagged SUMO conjugates from embryos**

Wild type Oregon-R embryos or embryos expressing tagged SUMO, were collected at 25^o^ C over a three-hour period, washed, incubated at 25 ^o^C or 37 ^o^C for an additional 45 minutes, then immediately frozen under liquid nitrogen. For anti-FLAG immunoprecipitation (IP), one gram of embryos was ground to a fine power under liquid nitrogen with a mortar and pestle, resuspended in 10 ml of TBST lysis buffer (50 mM Tris, pH 7.4, 300 mM NaCl, 1mM EDTA, 5% glycerol, and 1% Triton X-100), which was freshly supplemented with 50 mM N-ethylmaleimide (NEM, Sigma) and one Mini Complete Protease Inhibitor Cocktail Tablet (Roche). The embryos were further lysed by sonication and clarified by centrifugation at 32,000x g at 4 ^o^C for 10 min. The supernatant was incubated with 50 μl of mouse IgG agarose at 4 ^o^C for 30 min to remove proteins that non-specifically interact with IgG proteins and/or the agarose beads. The IgG beads were subsequently removed by centrifugation. The clarified supernatant was combined with 100 μl of anti-FLAG antibody conjugated agarose beads (Sigma), which had been blocked with 10% BCS TBST for 30 min, and gently mixed at 4 ^o^C for approximately 16 hours. Following the binding, the beads were pelleted, washed three times with cold TBST, and twice with TBS (TBST without Triton X-100). Finally, the bound proteins were eluted with 0.9 ml of 0.1 M glycine, pH 2.4, at 22 ^o^C for 10 min, immediately neutralized with 0.1 ml of 1 M Tris, pH 7.4, and 3 M NaCl, and precipitated with TCA/DOC (40 mg sodium deoxycholate in 100% w/v trichloroacetic acid). The precipitated protein pellet was resuspended in 20 μl of SDS loading buffer (63 mM Tris, pH 6.8, 10% glycerol, 0.1 M DTT, and 0.005% bromophenol blue), and were separated on SDS-PAGE and visualized by Sypro Ruby protein staining.

Protein purified by this protocol exhibited similar SDS-PAGE profiles (Figure S2) regardless of whether or not the embryos were subject to heat shock prior to lysis. From the heat-shocked sample, we identified a total of 319 proteins, and 276 proteins from the embryos not subjected to the heat-shock treatment, 234 proteins were common between the two sets of proteins. When we compared the numbers of peptides detected for these 234 proteins, we found that on average, 1.8 more peptides identified per protein for the heat-shocked sample (average peptides/protein = 7.59) than for sample not subjected to heat shock (average peptides/protein = 5.76). Among the 85 proteins that were uniquely found in the heat-shocked sample, 55 proteins were identified with 2 or fewer peptides and 66 proteins with 3 or fewer peptides. It is therefore likely that some of the 85 proteins that we failed to identify in the sample not subjected to heat shock were present in that sample at a level below our detection limit.

**Oligonucleotides**

| **Primer** | **Sequence** | **Description** |
| --- | --- | --- |
| O046_Smt3-f-NotIH6 | ACATCGCGGCCGCCATGGGTCACCATCACCACCACCATGATTACAAGGACGATGACGAT | Forward primer for PCR SUMO ORF, adding H_6_FLAG-tag and NotI site |
| O047_ Smt3-f-FLAG | CACCATGATTACAAGGACGATGACGATAAATCTGACGAAAAGAAGGGAGGTGAGACC | Forward primer for PCR SUMO ORF (overlapping O046) |
| O048_Smt3-r-XbaI | TCAGGTCTAGATTATGGAGCGCCACCAGTCTGCTG | Reverse primer for PCR SUMO ORF, adding XbaI site |
| O128_eIF4E5-ATG | GAAGTTATCAGTCGACATGGTAGTGTTGGAGACGGAGAAGACCAG | Forward primer for PCR eIF4E and cloning into pDNR-Dual |
| O129_eIF4E3-STOP | ATGGTCTAGAAAGCTTCTACAAAGTGTAGATCGATTTCACGTTGGAGC | Reverse primer for PCR eIF4E and cloning into pDNR-Dual |
| O171_1433-f | GAAGTTATCAGTCGACATGTCGACAGTCGATAAGGAAGAGCTGG | Forward primer for PCR 14-3-3 ζ and cloning into pDNR-Dual |
| O172_1433-r | ATGGTCTAGAAAGCTTTTAGTTGTCGCCGCCCTCCTGTG | Reverse primer for PCR 14-3-3 ζ and cloning into pDNR-Dual |
| O148_Ras1-K104R-f | GCAGATCAAGCGCGTAAGGGATGCCGAAGAGGTG | Sense strand of the primer pair for Ras1^K104R^ site-directed mutagenesis (sequence encoding mutated residue is underlined) |
| O149_Ras-K104R-r | CACCTCTTCGGCATCCCTTACGCGCTTGATCTGC | Anti-sense strand of the primer pair for Ras1^K104R^ site-directed mutagenesis (sequence encoding mutated residue is underlined) |
| O177_Rasv12-f | GTCGTTGGAGCCGTGGGCGTGGGCAAGTCC | Sense strand of the primer pair for Ras1^V12^ site-directed mutagenesis (sequence encoding mutated residue is underlined) |
| O178_Rasv12-r | GGACTTGCCCACGCCCACGGCTCCAACGAC | Anti-sense strand of the primer pair for Ras1^V12^ site-directed mutagenesis (sequence encoding mutated residue is underlined) |
| O200_Ras1-f | GAAGTTATCAGTCGACATGACGGAATACAAACTGGTCGTCGTTG | Forward primer for PCR Ras1 and cloning into pDNR-Dual |
| O213_Ras164r | ATGGTCTAGAAAGCTTTTAGCGGATTTCGCGCACCAGTG | Reverse primer for PCR Ras1^1-164^ and cloning into pDNR-Dual |
| O216_Ras7KR-r1 | GCGGTTCATACGTCTGCCCCTCCGCCCACGGTTGTCACGGTCGCGGCGGATTTCGCGCACCAG | Reverse primer 1 for mutating 7 lysines in Ras hypervariable region into arginines |
| O216_Ras7KR-r2 | ATGGTCTAGAAAGCTTTTAGAGCATTCTGCAACGAAATCTACGATTCGGGCGGTTCATACGTCTGCCCCT | Reverse primer 2 for mutating 7 lysines in Ras hypervariable region into arginines |
| O214_Ras165-f | GAAGTTATCAGTCGACAAGGACAAGGACAACAAGGGGCGGAGG | Forward primer for PCR Ras1^165-189^ and cloning into pDNR-Dual |
| O215_Ras189-r | ATGGTCTAGAAAGCTTTTAGAGCATTTTACATTTAAATCTACGATTCGG | Reverse primer for PCR Ras1 (including Stop codon) and cloning into pDNR-Dual |
| O130_Sqd5-ATG | GAAGTTATCAGTCGACATGGCCGAGAACAAGCAAGTGGATAC | Forward primer for PCR Squid and cloning into pDNR-Dual |
| O131_Sqd3-STOP | ATGGTCTAGAAAGCTTTTAGTAGGGCTGATGCCGCTGCTG | Reverse primer for PCR Squid and cloning into pDNR-Dual |
| O165_Polo-f | GAAGTTATCAGTCGACATGGCCGCGAAGCCCGAGG | Forward primer for PCR Polo and cloning into pDNR-Dual |
| O166_Polo-r | ATGGTCTAGAAAGCTTTTATGTGAACATCTTCTCCAGCATTTTCCTAAG | Reverse primer for PCR Polo and cloning into pDNR-Dual |
| T7-YFP-f | TAATACGACTCACTATAGGGAGAATGGTGAGCAAGGGCGAGGAGC | Forward primer for PCR YFP dsRNA template (T7 sequence underlined) |
| T7-YFP-r | TAATACGACTCACTATAGGGAGACTTGTACAGCTCGTCCATGCCGAGAG | Reverse primer for PCR YFP dsRNA template (T7 sequence underlined) |
| T7-SUMO-f | TAATACGACTCACTATAGGGAGATTTGACCACTTAGCAGCTTCAACAAGC | Forward primer for PCR SUMO dsRNA template (T7 sequence underlined) |
| T7-SUMO-r | TAATACGACTCACTATAGGGAGAACCATTTTCTTGTCTGCAAATGTTTTTG | Reverse primer for PCR SUMO dsRNA template (T7 sequence underlined) |

**SUPPLEMENTARY FIGURE LEGENDS**

**Figure S1. Detection of native and tagged SUMO in an anti-SUMO immunoblot.** H_6_-FLAG-SUMO (HF-SUMO) expression in early embryos was driven by a maternal-Gal4 driver. Wildtype Oregon-R embryos were used as the control. 0-3 hour embryo collections with or without 30 min heat shock at 37 ^o^C after collection were analyzed with anti-SUMO immunoblotting. The (His)_6_-FLAG-tag shifts the mobility of free SUMO, allowing distinction between the free tagged SUMO (open arrowhead) and the endogenous untagged SUMO (black arrowhead).

**Figure S2. FLAG-IP of embryos expressing tagged-SUMO or control embryos.** One-step purification by FLAG-IP was performed under identical conditions for all samples as described in the supplemental methods, and isolated proteins were separated and visualized by SDS-PAGE. 20 gel slices from each sample were subjected to tryptic digestion followed by LC-MS/MS. 1 μg of the BenchMark MW ladder (Invitrogen) and 7 μl of the Precision Plus Dual Color Marker were loaded onto each gel. Proteins were visualized with Sypro Ruby staining.

**Figure S3. *sumo P-*element mutant expresses reduced levels of *sumo* mRNA.** The *P*-element insertion allele of SUMO, *sumo^04493^,* expresses approximately 5-fold less *sumo* mRNA as assessed by semi-quantitative PCR of reverse transcribed total RNA when compared to Ore-R flies. The β-actin transcript was used for normalization.

**Figure S4. Bacterial sumoylation assays.** **A)** HP1, **B)** Squid, and **C)** Polo. Asterisks mark unmodified protein bands. Black arrows point to the bands representing sumoylated proteins. Open arrow indicates a non-specifically reacting band.

**Figure S5. Efficient knockdown of SUMO with dsRNA in larval tissues using a Gal4/UAS system**. *sumo* dsRNA was produced in the clonal patches (marked by the absence of CD2) where Act-Gal4 expression was induced. Wing imaginal disks were stained antibodies against CD2 (**A**, **B**) and SUMO (**A’**, **B’**), and with DAPI (**A”**, **B”**).

The arrowheads indicate the recombinant clones. **B-B”’**) Magnified view of the region outlined by the dashed line in **A”’**.
