# Supplementary figures and images for: Genetic and Proteomic Evidence for Roles of Drosophila SUMO in Cell Cycle Control, Ras Signaling, and Early Pattern Formation
Source: PLoS One. 2009 Jun 16;4(6):e5905. doi: 10.1371/journal.pone.0005905 (PMC2692000; doi:10.1371/journal.pone.0005905)

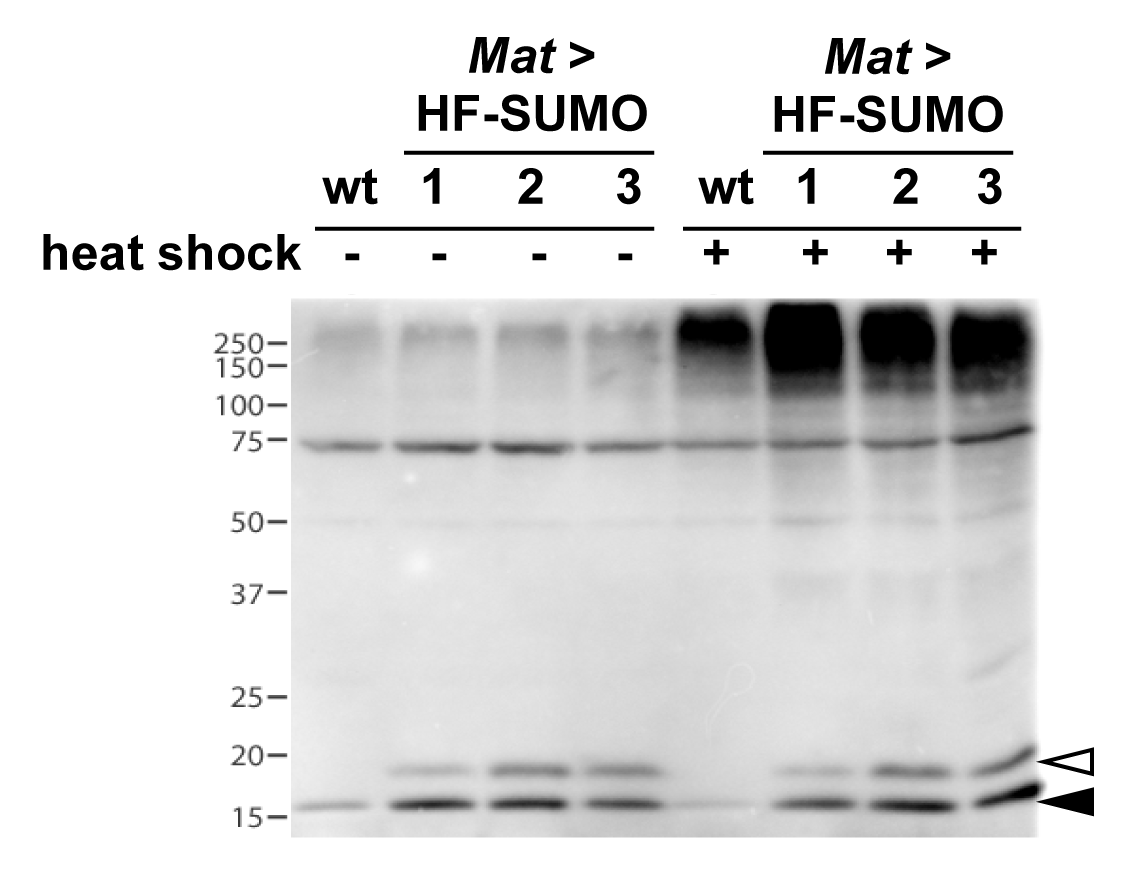

Supplement: Figure S1 — Detection of native and tagged SUMO in an anti-SUMO immunoblot (0.35 MB TIF) [file pone.0005905.s008.tif]

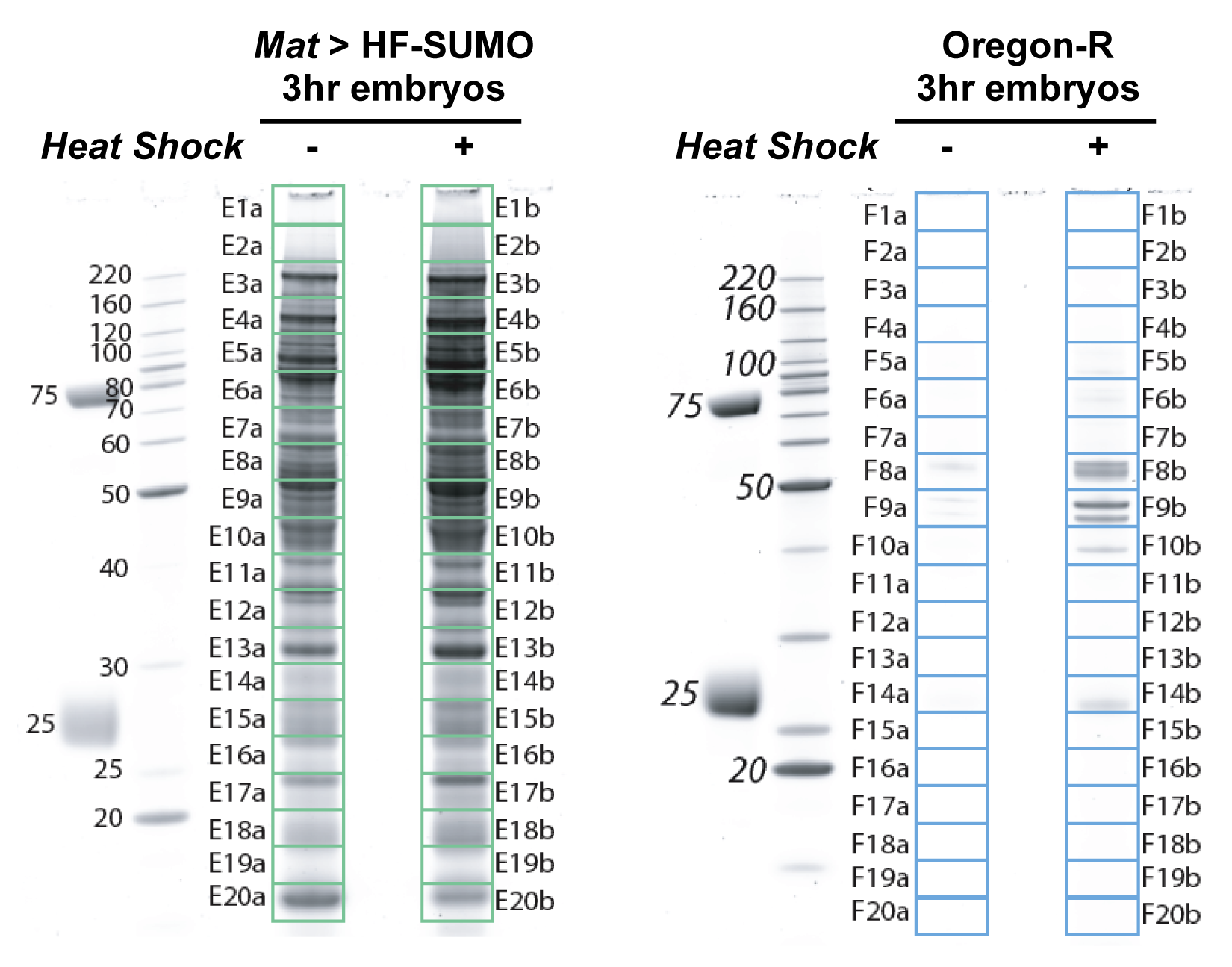

Supplement: Figure S2 — FLAG-IP of embryos expressing tagged-SUMO or control embryos (0.88 MB TIF) [file pone.0005905.s009.tif]

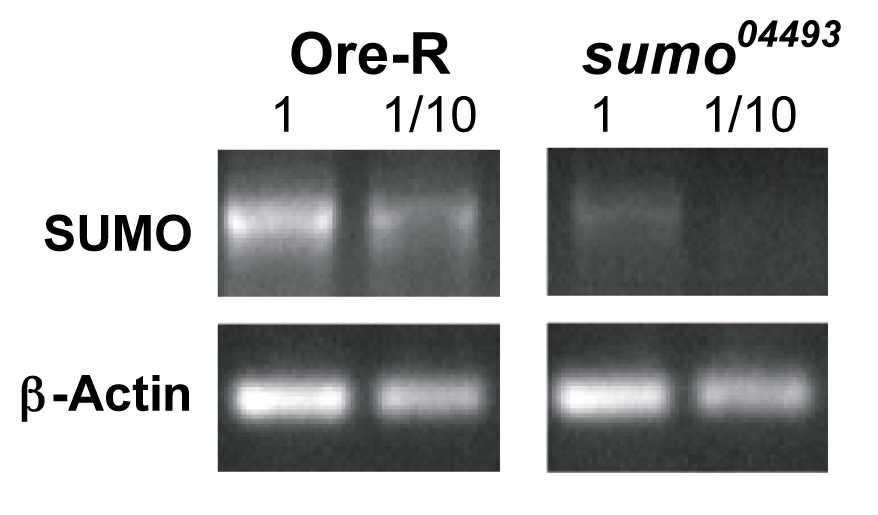

Supplement: Figure S3 — sumo P-element mutant expresses reduced levels of sumo mRNA (0.22 MB TIF) [file pone.0005905.s010.tif]

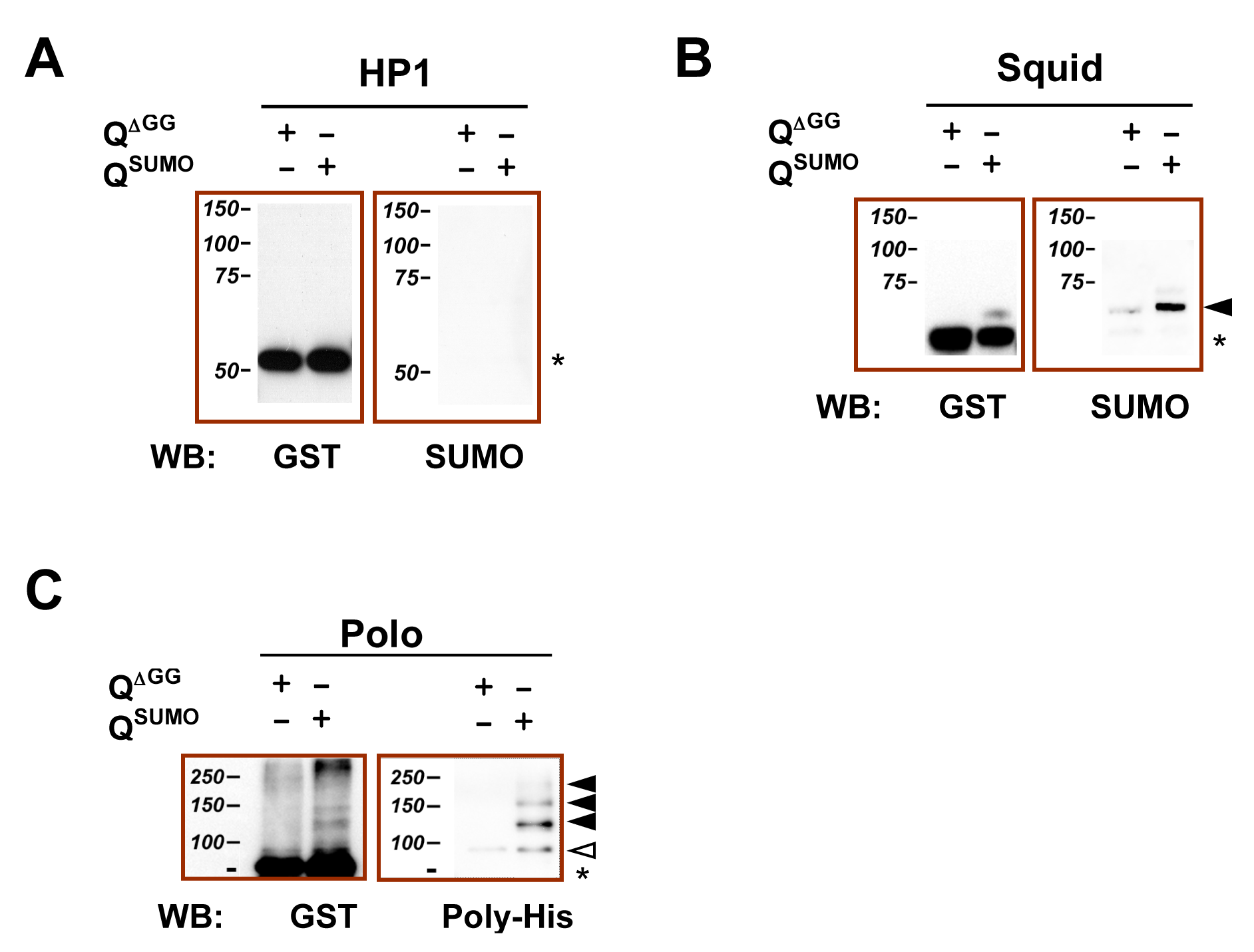

Supplement: Figure S4 — Bacterial sumoylation assays on HP1, squid, and Polo (0.50 MB TIF) [file pone.0005905.s011.tif]

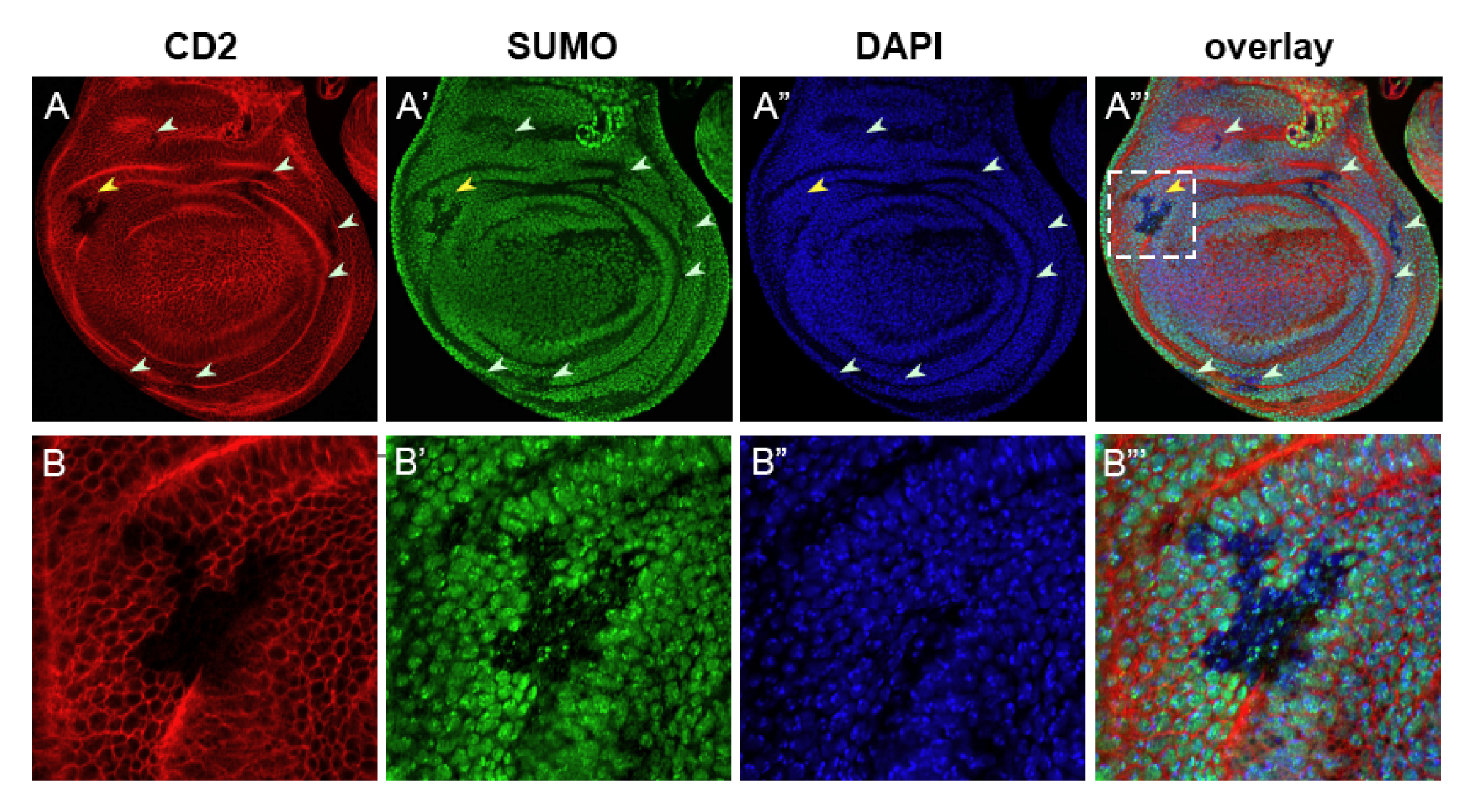

Supplement: Figure S5 — Efficient knockdown of SUMO with dsRNA in larval tissues using a Gal4/UAS system (3.20 MB TIF) [file pone.0005905.s012.tif]
